# The type of suture material affects transverse aortic constriction-induced heart failure development in mice: a repeated measures correlation analysis

**DOI:** 10.3389/fcvm.2023.1242763

**Published:** 2023-09-19

**Authors:** Benjamin Hackl, Eva Zabrodska, Stefanie Gewessler, Elena Lilliu, Eva Maria Putz, Attila Kiss, Bruno Podesser, Hannes Todt, Robin Ristl, Karlheinz Hilber, Xaver Koenig

**Affiliations:** ^1^ Department of Neurophysiology and Neuropharmacology, Center of Physiology and Pharmacology, Medical University of Vienna, Vienna, Austria; ^2^ Institute of Anatomy, First Faculty of Medicine, Charles University, Prague, Czech Republic; ^3^ St. Anna Children’s Cancer Research Institute (CCRI), Vienna, Austria; ^4^ Ludwig Boltzmann Institute for Cardiovascular Research at the Center for Biomedical Research and Translational Surgery, Medical University of Vienna, Vienna, Austria; ^5^ Center for Medical Statistics, Informatics and Intelligent Systems, Medical University of Vienna, Vienna, Austria

**Keywords:** TAC - transverse aortic constriction, suture material, heart failure, repeated measures correlation analysis, mice

## Abstract

**Introduction:**

Transverse-aortic constriction (TAC) operation is a widely used animal model to induce hypertrophy and heart failure through left-ventricular pressure overload. In mice, the cardiac response to TAC exhibits considerable variability influenced by factors such as strain, sub-strain, age, sex and vendor.

**Methods:**

To investigate the impact of suture material (silk versus prolene) and size (6-0 versus 7-0) on the TAC-induced phenotype, we performed surgeries on male C57BL6/N mice at 9 weeks of age defining the aortic constriction by a 27G needle, thereby employing most frequently used methodological settings. The mice were randomly assigned into four separate groups, 6-0 silk, 7-0 silk, 6-0 prolene and 7-0 prolene (10 mice per group). Echocardiography was conducted before TAC and every 4 weeks thereafter to monitor the development of heart failure. Repeated measures correlation analysis was employed to compare disease progression among the different groups.

**Results:**

Our findings reveal a significant influence of the chosen suture material on TAC outcomes. Mice operated with prolene showed increased mortality, slower body weight gain, faster left-ventricular mass increase, and a faster decline in left-ventricular ejection fraction, fractional shortening and aortic pressure gradient compared to silk-operated mice. Moreover, despite non significant, using thinner suture threads (7-0) tended to result in a more severe phenotype compared to thicker threads (6-0) across all tested parameters.

**Discussion:**

Collectively, our results highlight the importance of suture material selection in determining the cardiac phenotype induced by TAC and emphasize the need to consider this factor when comparing data across different research laboratories.

## 1. Introduction

Heart failure (HF) is defined as the inability of the heart to provide adequate blood flow and oxygen supply to meet the needs of the organism. HF commonly develops from different chronic cardiovascular diseases, including hypertension, coronary artery disease, arrhythmia and myocardial infarction. Transverse-aortic constriction (TAC) operation is a well-established model to induce hypertrophy and HF by left-ventricular (LV) pressure overload (1). It is generally accepted that the outcome of TAC in mice is subject to considerable variability within and across labs. In line, several factors that affect the TAC-induced phenotype have been reported. Comprehensively, the outcome of TAC operations is dependent on the size of the aortic constriction (2–4), which determines the remaining aortic diameter and the ensuing pressure gradient responsible for LV overload. However, respective cardiac hypertrophy and HF phenotype is also impacted by the specific strain (5, 6), sub-strain (7), parent-of-origin (8), and even vendor (5), as well as the age (9) and sex of the mice (8, 10). Furthermore, less apparent factors such as the surgical technician (2, 6, 8, 10–12) can impact TAC outcomes. Although a 27-gauge needle and silk suture material are commonly used to define the aortic constriction (13), other materials such as prolene are also frequently utilized. In the present study, we aimed to investigate whether variations in suture material (silk vs. prolene) and size (6-0 vs. 7-0) would impact TAC outcomes with respect to the HF phenotype. The suture thread size was defined following USP regulations, where size 6-0 and 7-0 is inversely correlated to a suture thread diameter of 0.085 and 0.06 mm, respectively (14). To track the development of HF, we used repeated measures correlation analysis, a form of ANCOVA (15–17), to not only assess the development of the population mean over time but also to respect the inter-individual variability between animals.

## 2. Material and methods

### 2.1. Animal housing

40 male C57BL/6 mice were purchased from Charles River, Germany. The substrain C57BL/6N was chosen for its increased vulnerability towards TAC operation (5, 7). Mice were housed under standard laboratory conditions in groups of 5 in GM500 cages within a DGM rack (Tecniplast) and provided with access to food (LASQCdiet®Rod16) and water ad libitum throughout the study. Mice were regularly monitored for health status, including visual inspection for signs of illness or injury. The facility followed appropriate hygiene protocols, such as regular cage cleaning and sterilization, to maintain a hygienic environment for the animals. These standardized conditions aimed to provide a consistent and controlled environment for the mice while ensuring their well-being and minimizing any potential confounding factors that could influence the study outcomes.

### 2.2. TAC surgery

Mice at the age of 8.8±0.2 weeks (mean±SD) were preoperatively anaesthetised by intraperitoneal injection with a mix of 0.3 mg/kg medetomidine, 1.0 mg/kg midazolam, 0.03 mg/kg fentanyl, and 10 mg/kg ketasol, and intubated using a 22G peripheral venous catheter (Venflon®, B.Braun, Germany) to allow mechanical ventilation of 175 μL lung volume at a rate of 150 breaths per minute. Eye ointment was applied to prevent dehydration. The mice were placed in supine position on a 37${}^{\circ }$C tempered heating plate and the extremities were fixed with adhesive tape. The upper thorax area was shaved and disinfected with povidone iodine. Median sternotomy followed by displacement of the thymus allowed access to the aortic arch. Animals were assigned into 4 separate groups (10 mice/group) to evaluate the effect of suture material and size on TAC. Polyfilament 6-0 silk (Perma-Hand™, K802H; Ethicon, Johnson & Johnson Medical N.V., Belgium) and 7-0 silk (SilkamR, DSMP7; B.Braun, Germany), as well as monofilament 6-0 polypropylen (Prolene™, 8711H; Ethicon) and 7-0 polypropylen (Prolene™, EH7405H; Ethicon) was used for TAC operation. According suture thread was placed around the aorta between the brachiocephalic trunk and the left common carotid artery and a square knot was tied against a blunted 27-gauge needle in order to create a reproducible degree of constriction for each animal. After removal of the gauge needle a third overhand knot was tied on top of the square knot. After successful ligation of the aortic arch and subsequent repositioning of the thymus, the sternum was secured with two stitches of 6-0 polysorb suture and the skin was closed with 4-0 polysorb suture and disinfected with povidone iodine. To antagonize anaesthesia, a mix of 1 mg/kg Antisedan® (atipamezole hydrochloride) and 1 mg/kg Anexate® (flumazenil) was injected. Then, 0.25 ml 1.6% Glucose in physiological NaCl solution was injected to compensate for blood loss during the surgery. After recovery from anesthesia (≈30 min) 0.06 mg/kg Temgesic® (buprenorphine) was injected and mice received a post-operative analgesic regimen of 0.12 mg/ml piritramide in 0.4% glucose supplemented drinking water ad libitum for 3 days.

### 2.3. Echocardiography

Echocardiography was performed prior and 4, 8, and 12 weeks after TAC operation. The mice were briefly (≈30 s) put in an anaesthesia box using 5% isoflurane, then placed in supine position with paws embedded in electrode gel and fixed with adhesive tape, that allowed recording of breathing frequency and ECG signal (lead II). Additionally, mouse body core temperature was measured with an anal probe. A heating plate (VisualSonics) and an infrared heating lamp was used to keep the body core temperature at $37\pm 0.5^{\circ }C$. Eye ointment was used to prevent dehydration. Hair removal cream was applied on the animal’s chest for 1 min. Echocardiography was performed under an isoflurane anaesthesia mask (1% in $O_{2}$ at 1.5 l/min), by a Vevo3100 preclinical imaging system (Fujifilm VisualSonics), using a MX250 transducer for imaging the aortic arch and a MX550D transducer for all other images. Using M-mode recordings in the short axis view of the heart (Figures 2A,B), we placed the scan line at the largest diameter in parallel to the papillary muscles to assess left ventricle inner diameter (LVID) as well as left ventricular thickness of the anterior (LVAW) and posterior wall (LVPW) during systole and diastole respectively. Analysis was done with the Vevo®Lab software (Fujifilm VisualSonics). Left ventricular end-diastolic volume (LVEDV) and end-systolic volume (LVESV) were calculated from LVID using the following formula: $7/(2.4+\mathrm{LVID})*{\mathrm{LVID}}^{3}$, with LVID derived under systolic and diastolic conditions, respectively. Ejection fraction (EF) was calculated as (LVEDV−LVESV)/LVEDV∗100. Corrected left ventricular mass (LVmass) was calculated as $1.053*[(\mathrm{LVID}+\mathrm{LVAW}+\mathrm{LVPW}{)}^{3}-{\mathrm{LVID}}^{3}]*0.8$ (18). To derive the pressure gradient at the constriction site we determined the maximum velocity by pulsewave Doppler mode at the aortic arch between the brachiocephalic trunc and the left common carotid artery. The pressure gradient (Δp) was calculated from the peak velocity (v) using a simplified Bernoulli equation $\Delta p=4v^{2}$ (19).

### 2.4. Statistical analysis

**Figure 1:**
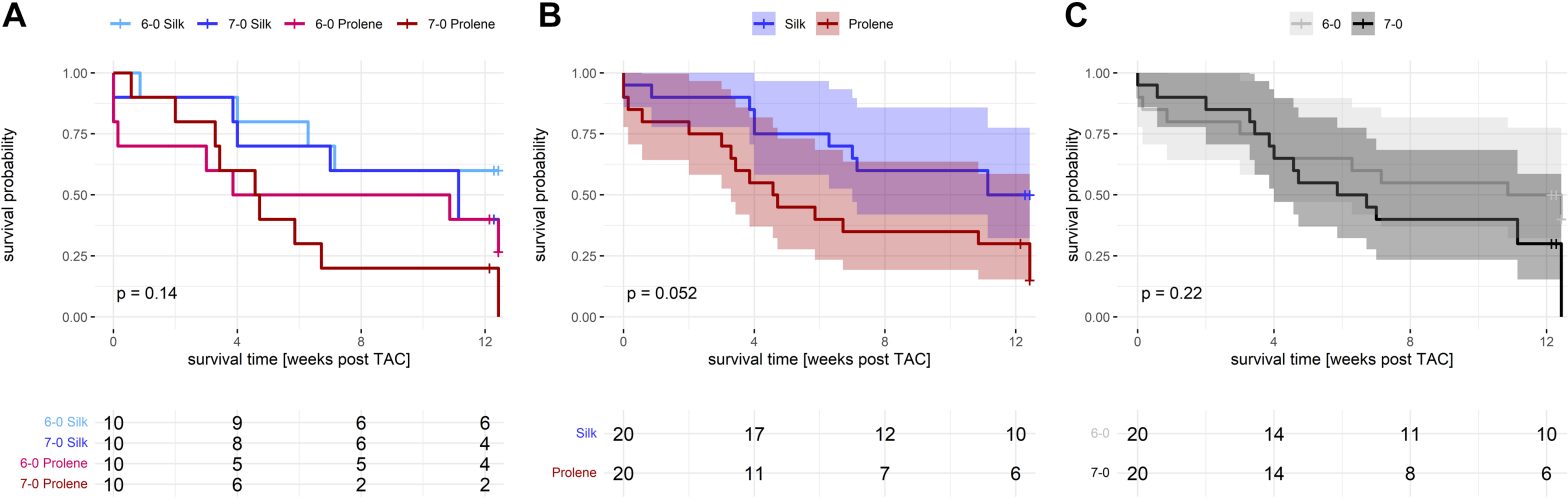
Effect of suture material and size on overall animal survival after TAC. (**A**) Separate Kaplan Meier plot including all 4 groups studied, 6-0 silk (light blue), 7-0 silk (dark blue), 6-0 prolene (light red), 7-0 prolene (dark red), for a followup period of 12 weeks after TAC. (**B**) Kaplan Meier plot comparing suture material (silk versus prolene) after pooling of 6-0 and 7-0 datasets. (**C**) Kaplan Meier plot comparing suture size (6-0 versus 7-0) after pooling of prolene and silk datasets. P-values were calculated using a log rank test.

All data were analyzed using R; respective source code can be found in the supplementary material. Kaplan-Meier survival analysis (Figure 1) was done using the “survminer” package, p-values from log-rank test results are displayed in the respective graphs. Risk tables below the Kaplan-Meier plots account for the amount of animals alive at time point 0, 4, 8 and 12 weeks post TAC operation. Repeated measures correlation analysis (RMCA; Figures 4–7) and in the supplementary material was performed in R, using the “rmcorr” package provided in Bakdash et al. (15). Individual animals were assigned a unique ID, and respective data are shown as dots in the RMCA graphs, with a dotted line connecting data points of the same animal (linear RMCA fit). Individual fits share the slope factor but differ in their ordinate intercept. Additionally, the repeated measures correlation coefficient $r_{rmc}$, p-value and slope factor are displayed in each RMCA analysis graph. Slope factors of different groups were compared using a z-test. For the comparison of silk versus prolene, 6-0 and 7-0 sizes were pooled, and similarly silk and prolene data were pooled in the comparison of 6-0 versus 7-0 material. No statistical difference was found between these groups regarding the HF parameters LVEF (Figure S8) and LVmass (Figure S9). A full analysis of all four separate groups (6-0 silk, 7-0 silk, 6-0 prolene, 7-0 prolene) can be found in the supplementary material.

### 2.5. Ethical considerations

The animal housing and experimental procedures adhered to the ethical guidelines outlined, have been approved by the animal welfare committee of the Medical University of Vienna and are covered by licence 2020-0.432.224 to Attila Kiss and Xaver Koenig issued by the Federal Ministry of the Republic of Austria.

## 3. Results

To investigate the impact of suture material on pressure overload-induced HF development we performed TAC operations on 40 male, adult C57BL/6N mice using four different kinds of suture materials, 6-0 silk, 7-0 silk, 6-0 prolene and 7-0 prolene (10 mice per group). Apart from the suture material, animals were treated similarly in all other aspects. We selected the C57BL/6N substrain for its increased susceptibility to TAC-induced HF development (7), thereby increasing the sensitivity to detect a potential impact of the suture materials studied. From the 40 operated animals, 6 died during or within 1 week after surgery accounting for 15% mortality in the initial phase after TAC. This incidence was higher than what we normally experience when using silk suture only. Taking a closer look revealed that the increase in mortality dominantly occurred in the 6-0 prolene operated animals (3 dead animals versus only 1 in every other group). In line, the overall survival after TAC revealed an increased incidence of death when comparing prolene to silk operated animals (Figures 1A,B). This effect just failed significance (p=0.052). Additionally, the groups operated with the thinner threads (7-0 silk and 7-0 prolene) demonstrated a trend towards increased mortality (Figures 1A,C). For comparison, no loss of animals occurred in a comparable cohort of n=8 SHAM-operated mice. Of note, due to the relatively strong HF phenotype of the 6N substrain a significant loss of animals was observed during the 12 week study period (Figure 1), in particular affecting the 7-0 prolene group. As a consequence, subsequent analysis of heart failure development by echocardiography was dominated by animals with a less severe cardiac phenotype and by data obtained at earlier time points.

**Figure 2:**
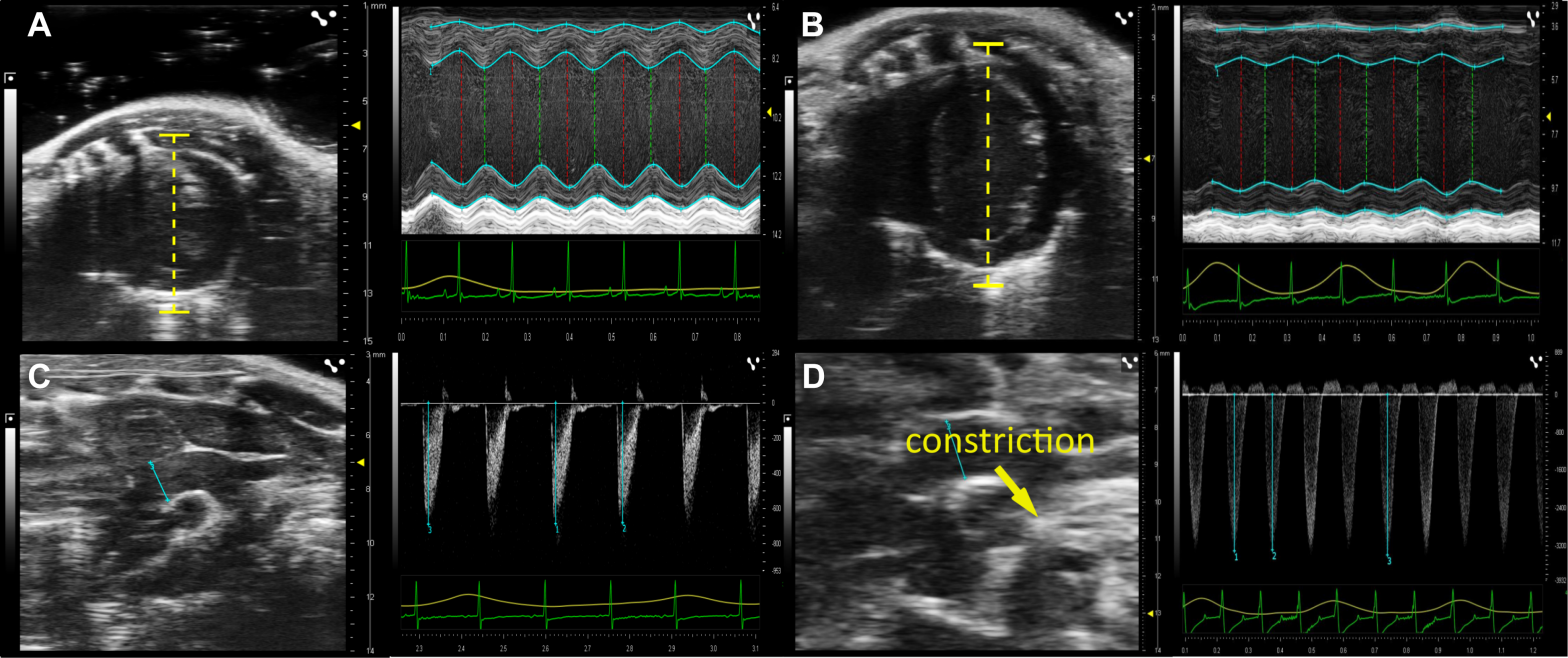
Development of heart failure after TAC operation. Original echocardiographic images before and 12 weeks after TAC. (**A**) Short Axis View (SAX) of the left ventricle (left image) and line scan (right image) measuring wall thickness and heart volume during systole and diastole before TAC surgery (time point 0) and (**B**) 12 weeks post TAC surgery. (**C**) Aortic Arch View (AA; left image) and pulsewave doppler velocity measurement of the transverse aortic arch (in mm/s) before TAC surgery and (**D**) 12 weeks post TAC surgery.

To monitor the progression of HF development we performed echocardiography before and 4, 8, and 12 weeks after TAC operation. Exemplary echocardiographic images before and 12 weeks after TAC are shown in (Figure 2). From respective recordings we obtained the left ventricular fractional shortening (FS) as a measure of systolic function, the left ventricular mass (LVmass) as a measure of cardiac hypertrophy and the pressure gradient (Δp) as a measure for the induced pressure overload upon aortic constriction.

**Figure 3:**
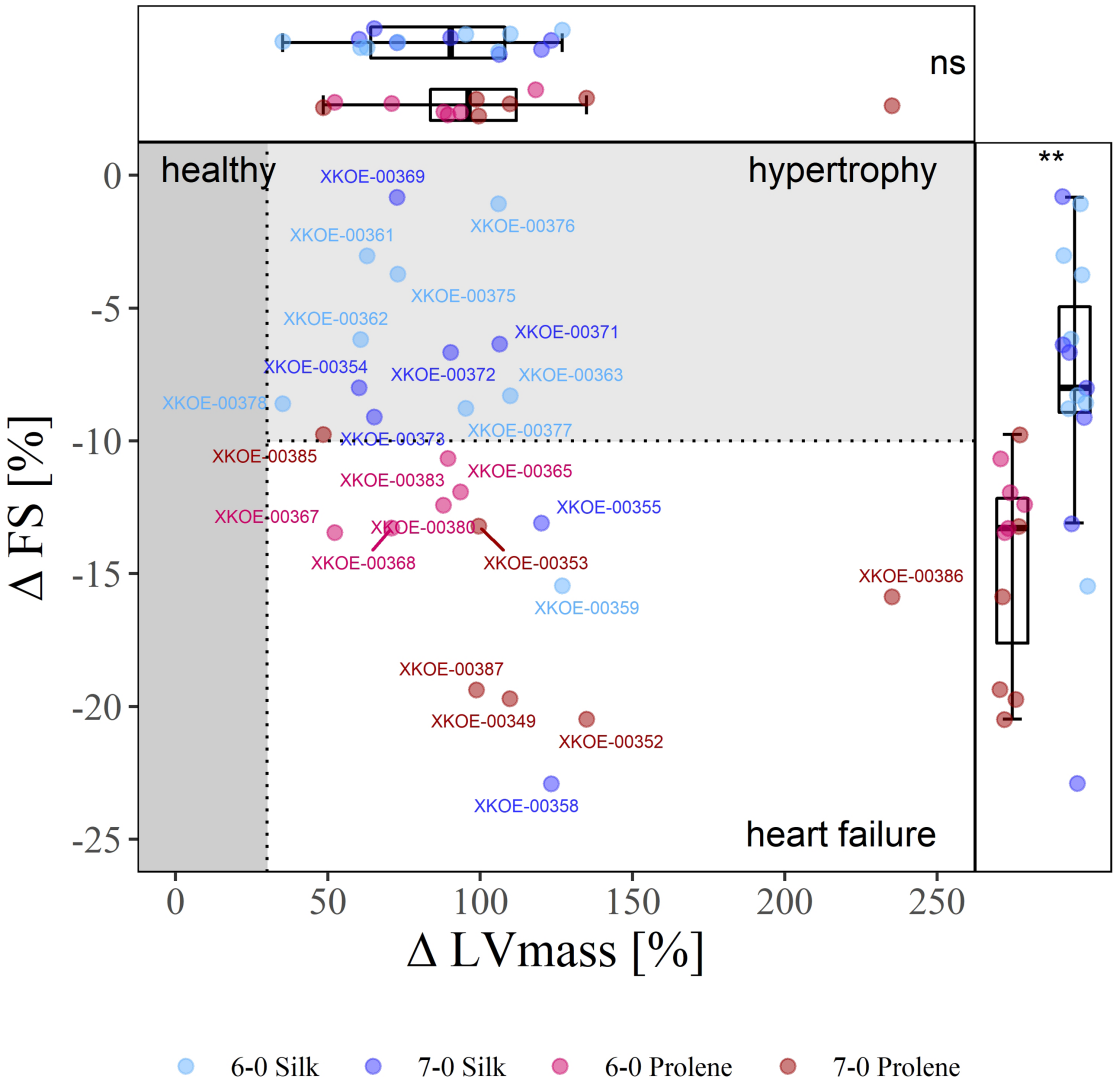
Effect of suture material and size on the severity of TAC-induced cardiac phenotype. 2D-Plot of the percentile change of FS and LVmass of all operated mice, 6-0 silk (light blue), 7-0 silk (dark blue), 6-0 prolene (light red), 7-0 prolene (dark red), derived from echocardiographic measurements before and 4 weeks after TAC. The phenotype domains “healthy” (dark grey), “hypertrophy” (light grey), and “heart failure” (white) were defined by an increase of more than 30% in LVmass and a decrease by more than 10% in FS. Each data point reflects an individual animal with respective animal-ID provided. Box plots with median, 95 % percentile and min max values of percentile change of FS and LVmass is shown at the right and top, respectively.

We first assessed the severity of TAC-induced HF development by comparing echocardiographic measurements before and 4 weeks after TAC. Based on the difference in FS and LVmass we defined three categories: (i) an overt increase in LVmass by more than 30% was considered to reflect hypertrophy, (ii) an additional decrease in FS by more than 10% was considered an early marker for the development of HF with reduced ejection fraction (HFrEF); we defined these thresholds in the absence of reports on respected criteria in mice regarding changes in LVmass and FS. (iii) all other animals were considered healthy. Along this classification a substantial clustering was observed for the different suture materials tested. Thus, Figure 3 shows a clear separation when comparing silk- and prolene-operated animals along the ΔFS−ΔLVmass plot, with silk-operated mice (blue) distributed preferentially towards the hypertrophy domain, while prolene operated mice (red) distributed preferentially towards the HF domain.

To test how fundamental phenotypic parameters developed and how they were influenced by the chosen suture material and size, we monitored HF development for 12 weeks after TAC and evaluated respective findings using repeated measures correlation analysis (RMCA; (15)). Here, RMCA provides a way to not only respect the temporal development of a specific parameter but also to incorporate the variability of individual animals. It does so by fitting a linear model with an individual intercept for each animal but a common slope and error variance for all animals.

**Figure 4:**
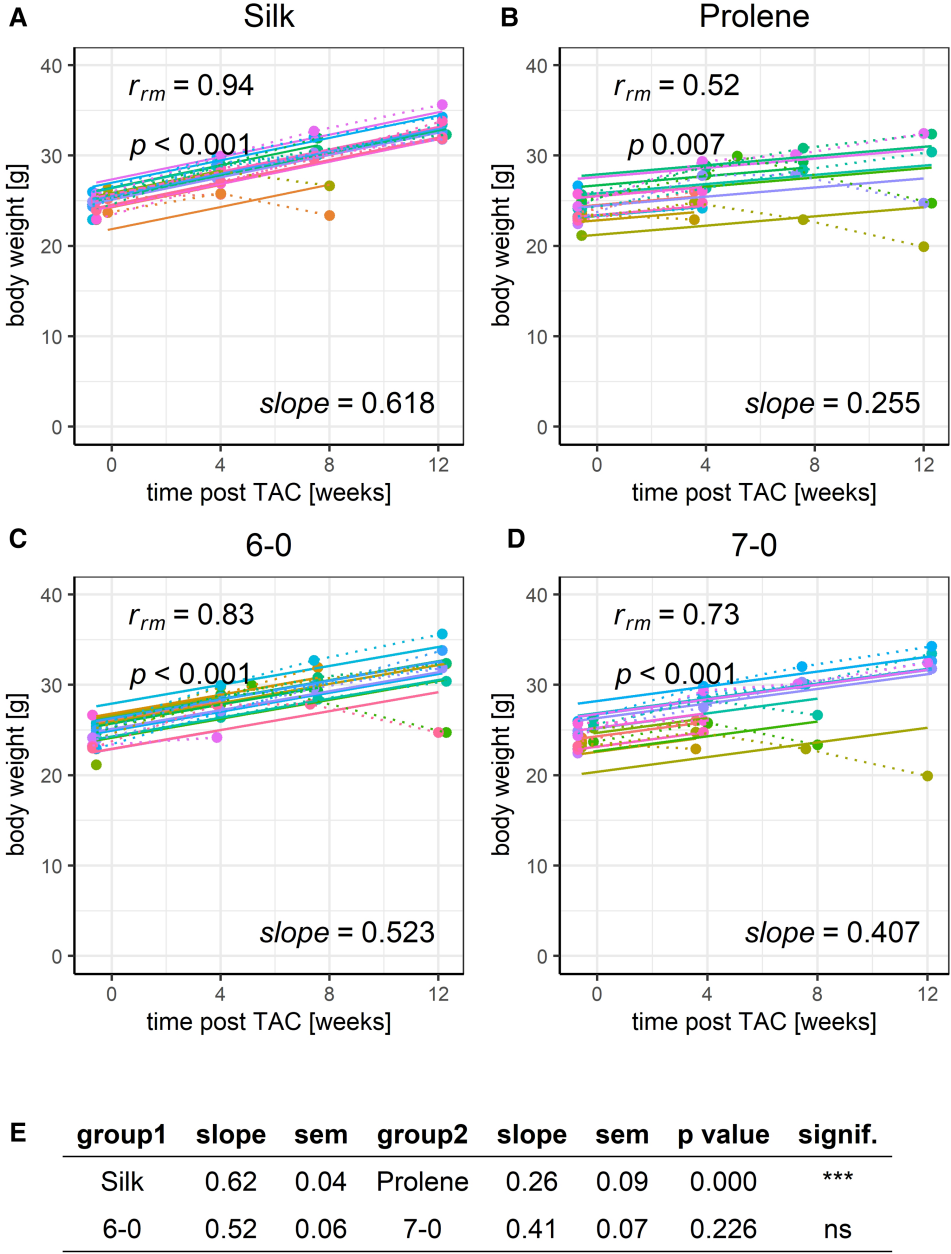
Effect of suture material and size on the development of body weight after TAC. Development of animal body weight within a 12-week time period after TAC for mice operated using different suture material, silk (**A**) or prolene (**B**), or using different suture size, 6-0 (**C**) or 7-0 (**D**), dotted line indicates individual animal progression, straight line indicates the RMCA fit for each animal. (**E**) z-score statistic of the comparison of the RMCA slopes for the pooled data groups silk (silk 6-0 and silk 7-0) versus rolene (prolene 6-0 and prolene 7-0) in the first row and 6-0 (silk 6-0 and prolene 6-0) versus 7-0 (silk 7-0 and prolene 7-0) in the second row. Slope mean values ± SEM are given.

With this tool at hand, we first analysed the effect of different suture material and size on the development of body weight after TAC (Figure 4). This parameter increased consistently with a 12-week period after TAC and was well fit with a linear model reflecting the reported normal body growth of this strain of mice. Data points deviating from the linear model occurred typically only at the terminal study endpoint when animals suddenly died or had to be sacrificed based on pre-defined ethical criteria. Such a decrease in body weight may correlate with the TAC-induced HF phenotype, which was also observed by others (5). The increase in body weight was less pronounced when animals were operated with prolene compared to silk, but no difference was observed when comparing different suture size, 6-0 versus 7-0 (Figure 4E). Body weight of mice assessed before TAC operation was not different across groups (Figure S1) indicating a homogeneous study population.

**Figure 5:**
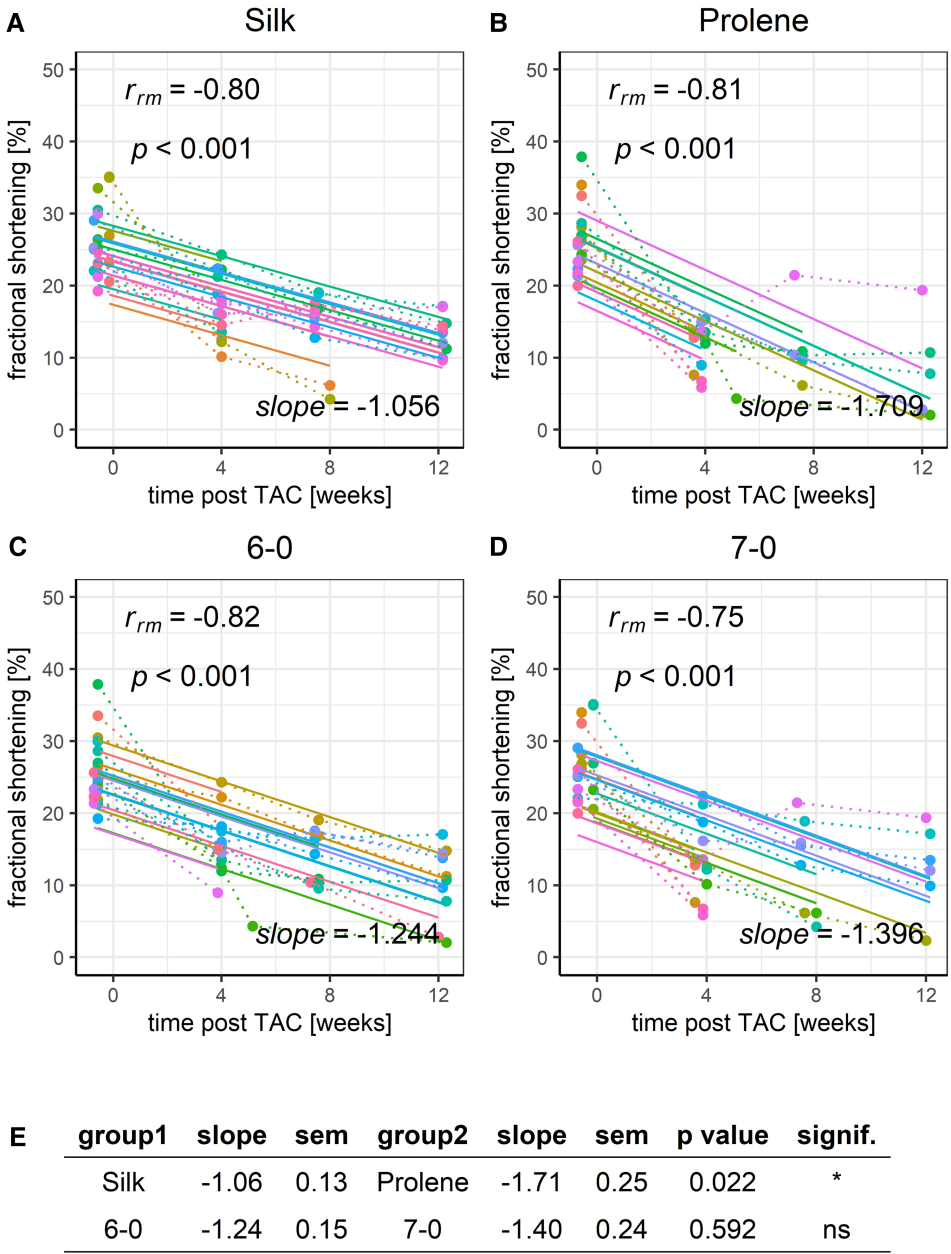
Effect of suture material and size on the development of fractional shortening after TAC. Development of animal FS within a 12-week time period after TAC for mice operated using different suture material, silk (**A**) or prolene (**B**), or using different suture size, 6-0 (**C**) or 7-0 (**D**), dotted line indicates individual animal progression, straight line indicates the RMCA fit for each animal. (**E**) z-score statistic of the comparison of the RMCA slopes for the pooled data groups.

We next applied RMCA to the development of FS (Figure 5) and observed a gradual decline in the FS for all groups tested, in full agreement with the development of a HF phenotype. The same was true for LVEF derived from long-axis images, Figure S10. However, the decline was more pronounced (decreased slope) when comparing prolene to silk operated animals (Figures 5A,B,E), while no difference in the slope was observed when comparing animals operated with suture 6-0 and 7-0 (Figures 5C–E). ANOVA analysis could only partly resolve the difference in FS in silk and prolene-operated animals (Figure S2). FS values before TAC operation were not different across tested groups (Figure S2) demonstrating that no bias was introduced when the different groups were assigned.

**Figure 6:**
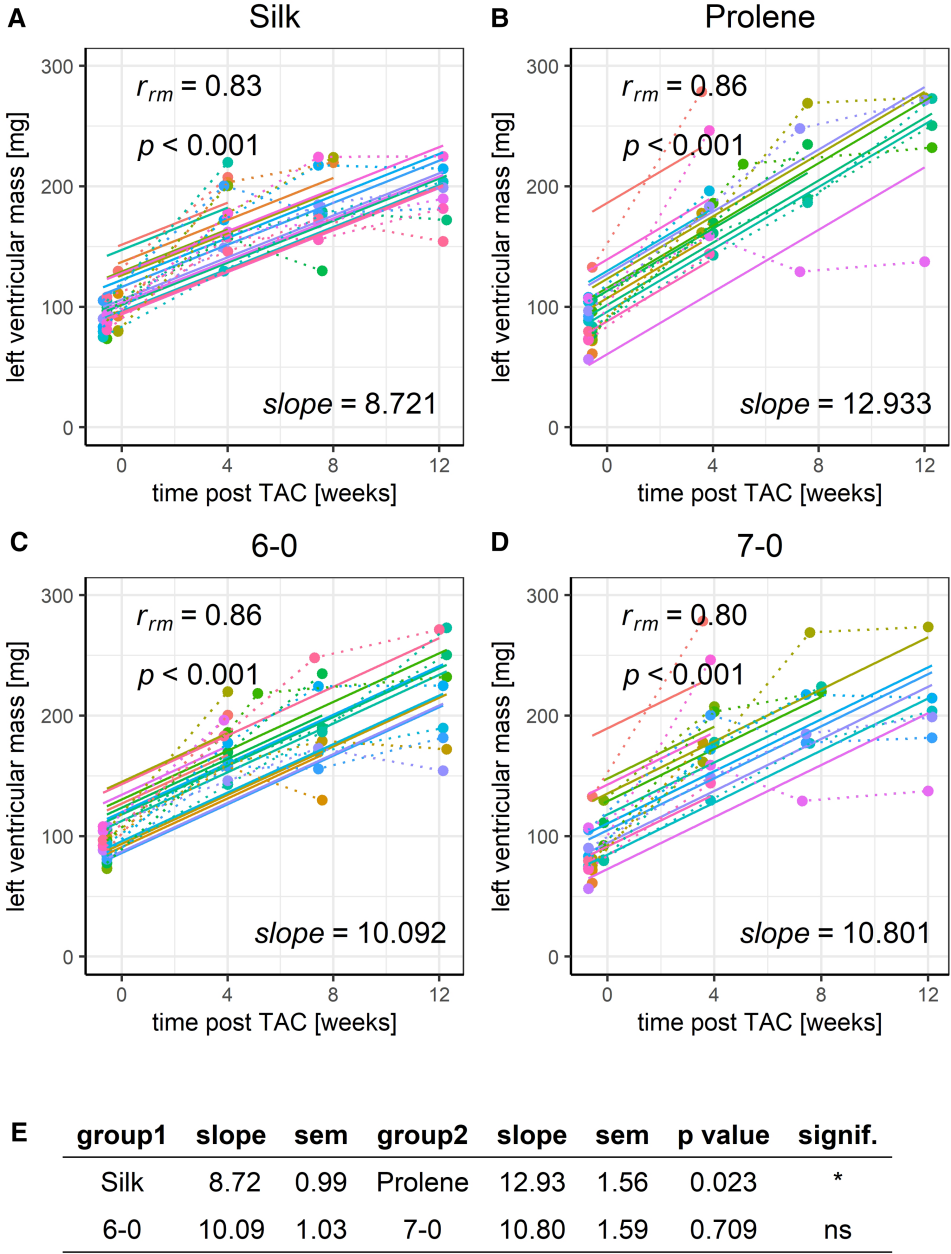
Effect of suture material and size on the development of left ventricular mass after TAC. Development of animal LVmass within a 12-week time period after TAC for mice operated using different suture material, silk (**A**) or prolene (**B**), or using different suture size, 6-0 (**C**) or 7-0 (**D**), dotted line indicates individual animal progression, straight line indicates the RMCA fit for each animal. (**E**) z-score statistic of the comparison of the RMCA slopes for the pooled data groups.

The same was true regarding LVmass (Figure 6), which increased substantially over the covered 12-week time period, reflecting a strong hypertrophy caused by the TAC-induced LV pressure overload. Prolene operated animals had a substantially faster gain of LVmass when compared to silk operated animals (Figures 6A,B,E), even more so when normalised to body weight (Figure S11). ANOVA analysis could only partly resolve the difference in LVmass in silk and prolene-operated animals (Figure S4) but demonstrated no difference in LVmass before TAC. Again, no difference was observed when comparing different suture sizes (Figures 6C–E).

**Figure 7:**
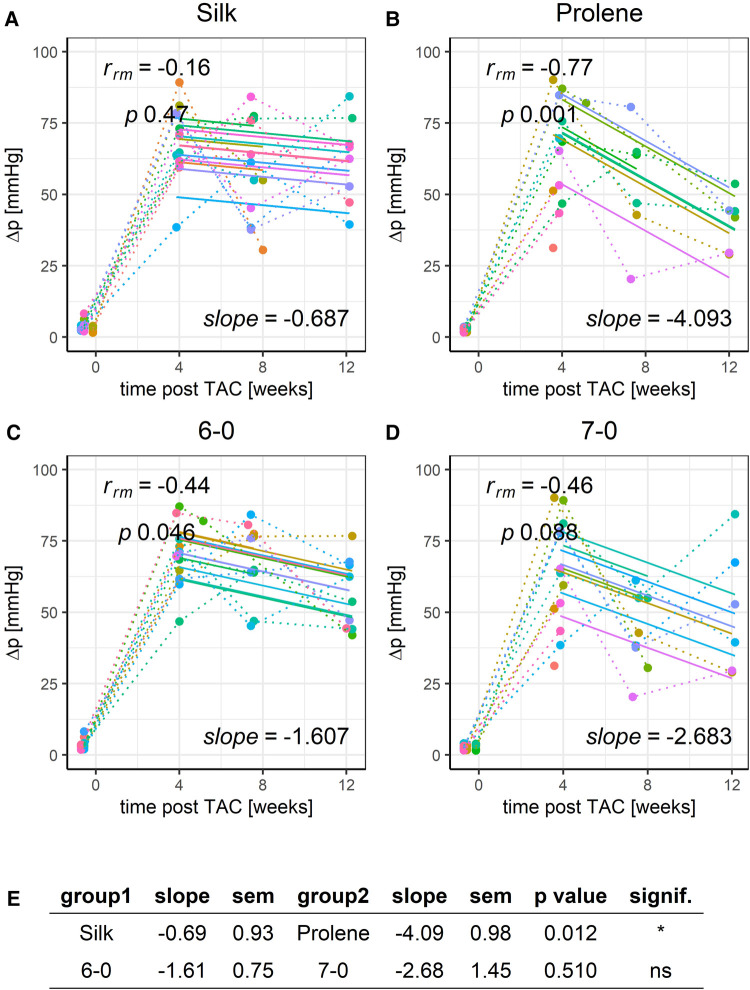
Effect of suture material and size on the development of pressure gradient after TAC. Development of animal Δp within a 12-week time period after TAC for mice operated using different suture material, silk (**A**) or prolene (**B**), or using different suture size, 6-0 (**C**) or 7-0 (**D**), dotted line indicates individual animal progression, straight line indicates the RMCA fit for each animal. (**E**) z-score statistic of the comparison of the RMCA slopes for the pooled data groups.

Finally, we looked at the pressure gradient (Δp), as calculated from the peak flow velocities before and after the constriction site. Δp values remained constant or slightly declined across the studied 12-week time period, reflecting a stable aortic constriction in all these animals. However, prolene operated animals had a more pronounced decline in Δp when compared to silk-operated animals (Figures 7A,B,E). This drop in pressure gradient values was not due to differences in cardiac output (Figure S12) or stroke volume (Figure S13) but may be explained by a reduction in contraction speed of the ventricular myocardium or a change of constriction diameter in the prolene-operated animals over time. A decline in pressure gradient over time in prolene-operated animals was also observed in another recent study (20). No difference was found when comparing suture size (Figures 7C–E).

Of note, one mouse (ID XKOE-00385) of the prolene 7-0 group seemed to develop no proper heart failure phenotype. A reduction in the FS and LVEF and an increase in LVmass seen after 4 weeks did not drop/increase further but even improved/dropped at 8 and 12 weeks post TAC and pressure gradient values followed the same pattern. A plausible explanation for this may be provided by a loosening of the aortic constriction after 4 weeks despite the greatest care in performing the TAC operations. We have decided to not exclude this animal from our analysis but obviously such an “outlier” will impact on the statistical evaluation. We therefore provide a separate RMCA excluding this animal in the supplementary material.

A list of numeric values for all evaluated echocardiographic parameters and a RMCA of SHAM-operated mice followed for the same time course are provided in Table 1 and Figure S14, respectively. The supplementary material also contains a non-pooled RMCA of BW (Figure S6), FS (Figure S7), LVEF (Figure S8) and LVmass (Figure S9) as well as an RMCA excluding mouse XKOE-00385, which did not develop a proper heart failure phenotype (Figures S15–S18).

## 4. Discussion

Here we have shown that the outcome of TAC operations is dependent on the type of suture material used. By employing repeated measure correlation analysis, a sensitive variant of ANCOVA (15–17), we demonstrated a significant difference in the development of hypertrophy and HF when using either silk or prolene suture for the aortic constriction. In particular, we observed that prolene-operated mice exhibited a significant reduction in body weight gain, a higher mortality, a faster increase in LVmass, as well as a faster reduction in FS when compared to silk-operated animals.

### 4.1. Suture material

In the past years, we have performed TAC-operations on over 500 animals employing 6-0 silk as our standard material. Silk stands out as the commonly preferred option (53%), while polyamide and prolene trail behind at (25%) and (12%), respectively (13). When compared to monofilament suture, braided material such as silk is generally stronger, more supple, easier to handle and better suited to tie a firm knot.

The size of the silk suture did not affect the overall handling and respective TAC outcome (Figure 1A). This was different for prolene, for which 6-0 was more difficult to handle than 7-0, and assessment of knot firmness proved more difficult despite a similar TAC outcome (Figure 1A). In a few animals the use of 6-0 prolene resulted in a tilting of the constriction knot after successful operation, which did not occur with any other suture material. The greater difficulties in handling 6-0 prolene and the potential to enforced constriction by knot rotation may explain the increased mortality seen in the first 24 h under those conditions. While we did not observe significant differences between suture size 6-0 and 7-0, we noticed a trend towards a more severe TAC-induced phenotype with the latter, thinner thread in all parameters tested. A potential effect may have escaped detection by a lack of statistical power. If indeed present it may be explained by a higher local pressure onto the aortic vessel with the thinner compared to the thicker thread. This may increase the propensity for local injury of the vessel endothelium and subsequent likelihood of plaque formation and inflammation, further aggravating the constriction. A post mortem investigation of respective parameters was however not performed but should be considered.

Given the contrasting survival rates and functional changes observed in animals operated with silk and prolene sutures throughout the study’s duration, however it is important to factor in additional considerations. Notably, the mechanical and elastic properties of prolene differ from those of silk, as highlighted in a study conducted by Naleway et al. (21). Thus, silk demonstrated a significantly higher elastic modulus compared to polypropylene and nylon thereby resulting in greater resistance to elongation upon load. While on the one hand this may allow for a more elastic constriction and thereby ease blood flow during systole for non-silk sutures, it may on the other hand narrow the intended constriction. The latter is due to the fact that a stretching of the suture during knot tying will relax from an elongated to a more constricted state once the knot is formed and the gauge needle removed. Given that Δp levels were comparable for all suture materials assessed at 4 weeks post TAC (Figure S5), this would imply that either these effects are small or may compensate for each other to a certain degree.

An obvious disadvantage of silk as a braided suture is its ability to soak up fluids and being a ground for bacteria in the gaps between the strands, which increases the risk of infections (22). In addition, natural suture materials such as silk consist of foreign proteins, that may trigger an immune response, leading to increased tissue reactivity, impaired wound healing, and increased scarring. However, as we observed a milder hypertrophy development and functional impairment for the silk and not for the prolene group, it is conceivable that altered immune response or changes in tissue reactivity around the constriction site did not account for the observed changes.

Our data add to the understanding that multiple factors have a direct and significant impact on the results of TAC operations in mice. Apart from previously identified factors, the choice of suture material introduces an additional variable that should be taken into account when employing this model. In addition, we also observed a trend of the different suture thread sizes to impact TAC outcome. If these and additional factors happen to coincide, the resulting differences in outcomes are likely to be substantial. This should become particularly relevant when comparing data across different research laboratories. It may also be worth to consider additional factors in this context that have not been explored to date. HF is associated with prominent alterations in myocardial metabolism and energy substrate utilisation (23–25). Consequently, the specific animal diet and housing conditions, including cage size, access to running wheels and similar devices, ambient temperature (26), light-dark cycle, and others may directly impact on TAC outcome. Another important factor to consider is the stress level of housed mice, which will impact on the sympathetic tone and circulating catecholamine levels (27). Undoubtedly, future studies will be necessary to address these aspects.

### 4.2. Repeated measures correlation analysis

Here we have employed Repeated Measures Correlation Analysis (RMCA), which is a form of ANCOVA (15–17). While usually ANCOVA is applied to test for the effect of a categorical independent variable on a continuous dependent variable by controlling for a second covariate, RMCA uses the underlying general linear model in a different way. It tests for the relationship between the two continuous variables (a dependent and an independent one; in our case, e.g., FS or LVEF and time after TAC), while controlling for an independent categorical variable (the individual animals, in our case). Thereby RMCA closely compares to a multilevel model, where a linear regression with random intercept but fixed slope is fit to account for dependencies within individual animals. Typically, random intercept models and the RMCA ANCOVA model give very similar estimates for the regression slope. However, in contrast to a mixed model, the RMCA ANCOVA model allows for a straight forward decomposition of the explained variance and thus allows for the calculation of a repeated measures correlation coefficient. An additional advantage of the approach pursued in this study is the fact that separate models are fit to each tested group. Hence, our analysis does not make an assumption of equal variances between groups. This assumption is often violated in usual ANCOVA models when there are different expected values between groups and variance typically scales with the expected value. Taken together, RMCA is less complex and easier to interpret than a random effects model with the drawback of being less flexible and not as easily scalable to more complex multi-level designs (15). Other common statistical methods such as Pearson or Spearman correlation cannot be applied as they assume independent observations.

### 4.3. Study limitations

The present study was conducted using the most commonly employed TAC parameters with respect to the age, strain, and sex of mice, as well as specific suture sizes, materials, and degree of constriction (13). This approach aimed to effectively control for potential confounding variables and ensure the relevance of our results for other research groups utilizing TAC operations as a model of HF. We deliberately opted for a relatively small sample size (n=10 per group), which is common in the field due to the significant effort associated with this model system. However, as a trade off of our rigorous methodology, our findings are limited to the effects of certain suture materials on male C57BL/6N mice of a specific age. Nonetheless, we believe that the effect of suture material on TAC outcome is still existent and relevant under more general conditions.

Another limitation is the fact that we cannot avoid exclusion bias caused by the early loss of animals in particular study groups. Thus, our results from the RMCA linear model describing the development of body weight, LVmass and LVEF are “dominated” by animals with a milder phenotype. With this bias, we consequently underestimate the true effect size. Also, we have assessed the TAC-induced phenotype solely by survival analysis and echocardiography in the present study. Consequently we do not know if fetal and fibrotic gene expression, LV tissue fibrosis and other established markers of this model are altered due to the chosen suture material. Finally, we want to mention that the haptic and optical differences in suture material precluded a blinding of the surgeon and that only the evaluation of the echocardiographic images was done in a blinded manner.

## Data Availability

The raw data supporting the conclusions of this article will be made available by the authors, without undue reservation.

## Supplementary material

ANOVA for all groups and time points

FIGURE S1

ANOVA of body weight. ANOVA was performed across all four tested groups at each time point (Echo0, before TAC; Echo1-3 at 4, 8 and 12 weeks post TAC) separately; respective p-values and n-numbers for the different groups at each time point are given at the top and bottom of each panel. Note that this analysis does not factor in the paired nature of the data as compared to RMCA.

FIGURE S2

ANOVA of FS. ANOVA was performed across all four tested groups at each time point (Echo0, before TAC; Echo1-3 at 4, 8 and 12 weeks post TAC) separately; respective p-values and n-numbers for the different groups at each time point are given at the top and bottom of each panel.

FIGURE S3

ANOVA of LVEF calculated from long axis evaluation. ANOVA was performed across all four tested groups at each time point (Echo0, before TAC; Echo1-3 at 4, 8 and 12 weeks post TAC) separately; respective p-values and n-numbers for the different groups at each time point are given at the top and bottom of each panel.

FIGURE S4

ANOVA of LVmass. ANOVA was performed across all four tested groups at each time point (Echo0, before TAC; Echo1-3 at 4, 8 and 12 weeks post TAC) separately; respective p-values and n-numbers for the different groups at each time point are given at the top and bottom of each panel.

FIGURE S5

ANOVA of Δp. ANOVA was performed across all four tested groups at each time point (Echo0, before TAC; Echo1-3 at 4, 8 and 12 weeks post TAC) separately; respective p-values and n-numbers for the different groups at each time point are given at the top and bottom of each panel. Note that pressure gradient was close to zero before TAC (Echo0) and strongly increased upon TAC (Echo1-3).

RMCA of non-pooled groups 6-0 silk, 7-0 silk, 6-0 prolene and 7-0 prolen

FIGURE S6

RMCA analysis of the development of body weight after TAC for all tested groups. Individual group of mice operated with either 6-0 silk (A), 7-0 silk (B), 6-0 prolene (C) and 7-0 prolene (D). Dotted lines indicate individual animal progression, straight lines indicate the RMCA fit for each animal. Respective slope factors with SEM and p-values as derived from a z-score statistic when comparing all groups across each other are given in E.

FIGURE S7

RMCA analysis of the development of FS after TAC for all tested groups. Individual group of mice operated with either 6-0 silk (A), 7-0 silk (B), 6-0 prolene (C) and 7-0 prolene (D). Dotted lines indicate individual animal progression, straight lines indicate the RMCA fit for each animal. Respective slope factors with SEM and p-values as derived from a z-score statistic when comparing all groups across each other are given in E.

FIGURE S8

RMCA analysis of the development of LVEF derived from long axis evaluation after TAC for all tested groups. Individual group of mice operated with either 6-0 silk (A), 7-0 silk (B), 6-0 prolene (C) and 7-0 prolene (D). Dotted lines indicate individual animal progression, straight lines indicate the RMCA fit for each animal. Respective slope factors with SEM and p-values as derived from a z-score statistic when comparing all groups across each other are given in E.

FIGURE S9

RMCA analysis of the development of LVmass after TAC for all tested groups. Individual group of mice operated with either 6-0 silk (A), 7-0 silk (B), 6-0 prolene (C) and 7-0 prolene (D). Dotted lines indicate individual animal progression, straight lines indicate the RMCA fit for each animal. Respective slope factors with SEM and p-values as derived from a z-score statistic when comparing all groups across each other are given in E.

RMCA of pooled groups silk, prolene, 6-0 and 7-0

FIGURE S10

Effect of suture material and size on the development of LVEF derived from long axis evaluation after TAC. Development of animal LVEF within a 12-week time period after TAC for mice operated using different suture material, silk (A) or prolene (B), or using different suture size, 6-0 (C) or 7-0 (D). Dotted line indicates individual animal progression, straight line indicates the RMCA fit for each animal. (E) z-score statistic of the comparison of the RMCA slopes for the pooled data groups. A RMCA of LVEF for all studied groups is provided in Fig.S8 and ANOVA of LVEF at all time poits studied provided in Fig.S3.

FIGURE S11

RMCA of left ventricular mass normalised to mouse body weight. Individual group of mice operated with either 6-0 silk (A), 7-0 silk (B), 6-0 prolene (C) and 7-0 prolene (D). Dotted lines indicate individual animal progression, straight lines indicate the RMCA fit for each animal. Respective slope factors with SEM and p-values as derived from a z-score statistic when comparing pooled silk versus prolene and 6-0 versus 7-0 groups are given in E.

FIGURE S12

RMCA of cardiac output. Individual group of mice operated with either 6-0 silk (A), 7-0 silk (B), 6-0 prolene (C) and 7-0 prolene (D). Dotted lines indicate individual animal progression, straight lines indicate the RMCA fit for each animal.

FIGURE S13

RMCA of stroke volume. Individual group of mice operated with either 6-0 silk (A), 7-0 silk (B), 6-0 prolene (C) and 7-0 prolene (D). Dotted lines indicate individual animal progression, straight lines indicate the RMCA fit for each animal. Respective slope factors with SEM and p-values as derived from a z-score statistic when comparing pooled silk versus prolene and 6-0 versus 7-0 groups are given in E.

Parameters of SHAM-operated animals

FIGURE S14

Body weight and functional parameters in SHAM operated animals. Temporal development and RMCA of body weight (A), left ventricular mass (B), fractional shortening (C) and pressure gradient (D) for a total of n = 8 SHAM operated animals. Apart from the aortic constriction SHAM operated mice received the same treatment and operation procedure as TAC-operated ones.

RMCA with animal XKOE-00385 excluded

Mouse (ID XKOE-00385) of the prolene 7-0 group seemed to develop no proper heart failure phenotype. A reduction in the FS and LVEF and an increase in LVmass seen after 4 weeks did not drop/increased further but even improved/dropped at 8 and 12 weeks post TAC and pressure gradient values followed the same pattern. A plausible explanation for this may be provided by a loosening of the aortic constriction after 4 weeks despite the greatest care in performing the TAC operations. While this animal is not excluded in the main body of the manuscript, we here provide a separate RMCA excluding this animal.

Figure S15

RMCA of LVmass excluding animal XKOE-00385. Individual group of mice operated with either 6-0 silk (A), 7-0 silk (B), 6-0 prolene (C) and 7-0 prolene (D). Dotted lines indicate individual animal progression, straight lines indicate the RMCA fit for each animal. Respective slope factors with SEM and p-values as derived from a z-score statistic when comparing all groups across each other are given in E.

FIGURE S16

RMCA of FS excluding animal XKOE-00385. Individual group of mice operated with either 6-0 silk (A), 7-0 silk (B), 6-0 prolene (C) and 7-0 prolene (D). Dotted lines indicate individual animal progression, straight lines indicate the RMCA fit for each animal. Respective slope factors with SEM and p-values as derived from a z-score statistic when comparing all groups across each other are given in E.

FIGURE S17

RMCA of LVEF derived from long axis evaluation excluding animal XKOE-00385. Individual group of mice operated with either 6-0 silk (A), 7-0 silk (B), 6-0 prolene (C) and 7-0 prolene (D). Dotted lines indicate individual animal progression, straight lines indicate the RMCA fit for each animal. Respective slope factors with SEM and p-values as derived from a z-score statistic when comparing all groups across each other are given in E.

FIGURE S18

RMCA of Δp excluding animal XKOE-00385. Individual group of mice operated with either 6-0 silk (A), 7-0 silk (B), 6-0 prolene (C) and 7-0 prolene (D). Dotted lines indicate individual animal progression, straight lines indicate the RMCA fit for each animal. Respective slope factors with SEM and p-values as derived from a z-score statistic when comparing all groups across each other are given in E.

TABLE S1

Cardiac function and ventricular dimensions as assessed by transthoracic echocardiography for all groups (6-0 silk, 7-0 silk, 6-0 prolene and 7-0 prolene) and at all time points (Echo 0, 1, 2 and 3). Data are displayed as mean ± SEM with n-numbers in brackets. Parameters in descending oder: SAX FS, fractional shortening from short axis evaluation; AA peak vel., aortic arch peak velocity; BW, body weight; CO, cardiac output; HR, heart rate; HW/BW, heart to body weight ratio; LVAW, left ventricular anterior wall thickness during diastole (d) or systole (s); LVmass, left ventricular mass; LVPW, left ventricular posterior wall thickness during diastole (d) or systole (s); p.grad, pressure gradient; PSLAX A, left ventricular inner area from parasternal long axis evaluation during diastole (d) or systole (s); PSLAX LVEF, left ventricular ejection fraction from long axis evaluation; PSLAX SV, stroke volume from parasternal long axis evaluation; PSLAX V, inner ventricular volume from parasternal long axis evaluation during diastole (d) or systole (s); RR, respiratory rate; SAX diam, left ventricular inner diameter from short axis evaluation during diastole (d) or systole (s); Respective parameter units are given in brackets.

DATA SHEET S1

R-scripts for the employed RMCA. Analysis was performed with RStudio 2022.12.0+353 “Elsbeth Geranium” Release (7d165dcfc1b6d300eb247738db2c7076234f6ef0, 2022-12-03) for Windows Mozilla/5.0 (Windows NT 10.0; Win64; x64) AppleWebKit/537.36 (KHTML, like Gecko) RStudio/2022.12.0+353 Chrome/102.0.5005.167 Electron/19.1.3 Safari/537.36. The following Rmd files were used to run the analyses and generate the figures: 1) SupplementaryCode-Echocardiography.Rmd, 2) SupplementaryCode-RepeatedMeasuresCorrelation.Rmd, 3) SupplementaryCode-SuitureSurvival.Rmd; RawData.xlsx file for data input needs the following column structure: → individual animal names, e.g. Mouse1, Mouse2, … → grouping variable, e.g. 6-0 Silk, 7-0 Silk, 6-0 Prolene, 7-0 Prolene, < date – echo > → in excel serial number date format, e.g. 1/1/2023 equals 44915, < date – surgery > → in excel serial number date format, then append all other parameters to be analysed.

## References

[1] Rockman HA Ross RS Harris AN Knowlton KU SteinhelperT ME FieldT LJ, et al. Segregation of atrial-specific, inducible expression of an atrial natriuretic factor transgene in an in vivo murine model of cardiac hypertrophy. Proc Natl Acad Sci USA (1991) 88(18):8277–81. 10.1073/pnas.88.18.8277 1832775 PMC52490

[2] Nagayama T Hsu S Zhang M Koitabashi N Bedja D Gabrielson KL, et al. Pressure-overload magnitude-dependence of the anti-hypertrophic efficacy of PDE5a inhibition. J Mol Cell Cardiol. (2009) 46(4):560–7. 10.1016/j.yjmcc.2008.12.008 19159628 PMC2703675

[3] Richards DA Aronovitz MJ Calamaras TD Tam K Martin GL Liu P, et al. Distinct phenotypes induced by three degrees of transverse aortic constriction in mice. Sci Rep. (2019) 9(1):5844. 10.1038/s41598-019-42209-7 30971724 PMC6458135

[4] Deng H Ma L-L Kong F-J Qiao Z. Distinct phenotypes induced by different degrees of transverse aortic constriction in C57BL/6N mice. Front Cardiovasc Med. (2021) 8:641272. 10.3389/fcvm.2021.641272 33969009 PMC8100039

[5] Garcia-Menendez L Karamanlidis G Kolwicz S Tian R. Substrain specific response to cardiac pressure overload in C57BL/6 mice. Am J Physiol Heart Circ Physiol. (2013) 305(3):H397–H402. 10.1152/ajpheart.00088.2013 23709599 PMC3742875

[6] Barrick CJ Rojas M Schoonhoven R Smyth SS Threadgill DW. Cardiac response to pressure overload in 129S1/SvImJ, C57BL/6J mice: temporal- and background-dependent development of concentric left ventricular hypertrophy. Am J Physiol Heart Circ Physiol. (2007) 292(5):H2119–30. 10.1152/ajpheart.00816.2006 17172276

[7] Nickel A von Hardenberg A Hohl M Löffler J Kohlhaas M Becker J, et al. Reversal of mitochondrial transhydrogenase causes oxidative stress in heart failure. Cell Metab. (2015) 22(3):472–84. 10.1016/j.cmet.2015.07.008 26256392

[8] Barrick CJ Dong A Waikel R Corn D Yang F Threadgill DW, et al. Parent-of-origin effects on cardiac response to pressure overload in mice. Am J Physiol Heart Circ Physiol. (2009) 297(3):1003–9. 10.1152/ajpheart.00896.2008 PMC2755989 19561308

[9] Geng X Hwang J Ye J Shih H Coulter B Naudin C, et al. Aging is protective against pressure overload cardiomyopathy via adaptive extracellular matrix remodeling. Am J Cardiovasc Dis. (2017) 7(3):72–82. 28695053 PMC5498818

[10] Skavdahl M Steenbergen C Clark J Myers P Demianenko T Mao L, et al. Estrogen receptor-β mediates male-female differences in the development of pressure overload hypertrophy. Am J Physiol Heart Circ Physiol. (2005) 288:H469–76. 10.1152/ajpheart.00723.2004 15374829

[11] Liao Y Ishikura F Beppu S Asakura M Takashima S Asanuma H, et al. Echocardiographic assessment of LV hypertrophy and function in aortic-banded mice: necropsy validation. Am J Physiol Heart Circ Physiol. (2002) 282(5):H1703–8. 10.1152/ajpheart.00238.2001 11959634

[12] Rothermel BA Berenji K Tannous P Kutschke W Dey A Nolan B, et al. Differential activation of stress-response signaling in load-induced cardiac hypertrophy and failure. Physiol Genomics. (2005) 23(1):18–27. 10.1152/physiolgenomics.00061.2005 16033866 PMC4118287

[13] Bosch L de Haan JJ Bastemeijer M van der Burg J van der Worp E Wesseling M, et al. The transverse aortic constriction heart failure animal model: a systematic review and meta-analysis. Heart Fail Rev. (2021) 26(6):1515–24. 10.1007/s10741-020-09960-w 32335789 PMC8510918

[14] Byrne M Aly A. The surgical suture. Aesthet Surg J. (2019) 39(Suppl. 2):S67–S72. 10.1093/asj/sjz036 30869751

[15] Bakdash JZ Marusich LR. Repeated measures correlation. Front Psychol. (2017) 8:456. 10.3389/fpsyg.2017.00456 28439244 PMC5383908

[16] Bland JM Altman DG. Statistics notes: calculating correlation coefficients with repeated observations: part 1–correlation within subjects. BMJ. (1995a) 310:446. 10.1136/bmj.310.6977.446 7873953 PMC2548822

[17] Bland JM Altman DG. Statistics notes: calculating correlation coefficients with repeated observations: part 2–correlation between subjects. BMJ. (1995b) 310:633. 10.1136/bmj.310.6980.633 7703752 PMC2549010

[18] Teichholz LE Kreulen T Herman MV Gorlin R. Problems in echocardiographic volume determinations: echocardiographic-angiographic correlations in the presence or absence of asynergy. Am J Cardiol. (1976) 37(1):7–11. 10.1016/0002-9149(76)90491-4 1244736

[19] Harris P Kuppurao L. Quantitative doppler echocardiography. BJA Educ. (2016) 16(2):46–52. 10.1093/bjaceaccp/mkv015

[20] Hermans H Swinnen M Pokreisz P Caluwé E Dymarkowski S Herregods M-C, et al. Murine pressure overload models: a 30-MHz look brings a whole new “sound” into data interpretation. J Appl Physiol. (2014) 117(5):563–71. 10.1152/japplphysiol.00363.2014 25059236

[21] Naleway SE Lear W Kruzic JJ Maughan CB. Mechanical properties of suture materials in general and cutaneous surgery: an update on mechanical properties of suture materials. J Biomed Mater Res B Appl Biomater. (2015) 103(4):735–42. 10.1002/jbm.b.33171 25045025

[22] Togo S Kubota T Takahashi T Yoshida K Matsuo K Morioka D, et al. Usefulness of absorbable sutures in preventing surgical site infection in hepatectomy. J Gastrointest Surg. (2008) 12(6):1041–6. 10.1007/s11605-007-0297-6 17899302

[23] Neubauer S. The failing heart—an engine out of fuel. N Engl J Med. (2007) 356(11):1140–51. 10.1056/NEJMra063052 17360992

[24] Doenst T Nguyen TD Abel ED. Cardiac metabolism in heart failure. Circ Res. (2013) 113(6):709–24. 10.1161/CIRCRESAHA.113.300376 23989714 PMC3896379

[25] Kato T Niizuma S Inuzuka Y Kawashima T Okuda J Tamaki Y, et al. Analysis of metabolic remodeling in compensated left ventricular hypertrophy and heart failure. Circ Heart Fail. (2010) 3(3):420–30. 10.1161/CIRCHEARTFAILURE.109.888479 20176713

[26] Chen X Bollinger E Cunio T Damilano F Stansfield JC Pinkus CA, et al. An assessment of thermoneutral housing conditions on murine cardiometabolic function. Am J Physiol-Heart and Circulatory Physiology. (2022) 322(2):H234–45. 10.1152/ajpheart.00461.2021 34919456

[27] Rau CD Wang J Avetisyan R Romay MC Martin L Ren S, et al. Mapping genetic contributions to cardiac pathology induced by beta-adrenergic stimulation in mice. Circ Cardiovasc Genet. (2015) 8(1):40–9. 10.1161/CIRCGENETICS.113.000732 25480693 PMC4334708

