# Supplementary material for: The type of suture material affects transverse aortic constriction-induced heart failure development in mice: a repeated measures correlation analysis
Source: Front Cardiovasc Med. 2023 Sep 19;10:1242763. doi: 10.3389/fcvm.2023.1242763 (PMC10546326; doi:10.3389/fcvm.2023.1242763)
Supplement: Supplementary file 1 [file Datasheet1.docx]

**SupplementaryCode-Echocardiography.Rmd**

---

title: "The type of suture material affects transverse aortic constriction induced heart failure development in mice: A repeated measures correlation analysis"

author: "Benjamin Hackl"

date: "`r format(Sys.Date(), '%d.%m.%Y')`"

output:

pdf_document:

toc: false

number_sections: true

toc_depth: 2

df_print: kable

highlight: tango

latex_engine: xelatex

mainfont: "Calibri"

monofont: "Calibri Light"

---

```{r, include=FALSE, warning=FALSE}

rm(list=ls()) # clean your workspace

# list of required packages

.packages = c("here", "googlesheets4", "xlsx", "openxlsx", "readxl", "writexl", "tidyr", "plyr", "dplyr", "purrr", "ggplot2", "ggpubr", "ggpmisc", "ggrepel", "ggthemes", "ggpval", "ggforce", "cowplot", "lmerTest", "rstatix", "svglite","survminer", "survival", "RTCGA.clinical", "coxme", "gridExtra", "scales")

# install CRAN packages (if not already installed)

.inst <- .packages %in% installed.packages()

if(length(.packages[!.inst]) > 0) install.packages(.packages[!.inst])

# load packages into session

lapply(.packages, require, character.only=TRUE)

```

```{r, include=FALSE, warning=FALSE}

setwd(dirname(rstudioapi::getActiveDocumentContext()$path))

RawData <- read_excel("./RawData.xlsx", sheet= 2, na = "")

RawData <- RawData[(RawData$series == "SS3"),]

RawData <- RawData %>% drop_na(Animal_ID)

RawData$group <- factor(RawData$group , levels=c("6-0 Silk", "7-0 Silk", "6-0 Prolene", "7-0 Prolene"))

################################################################################

filename <- tools::file_path_sans_ext(basename(rstudioapi::getActiveDocumentContext()$path))

Ranalysis_path <- paste0("./RESULTS.", filename)

if (!dir.exists(Ranalysis_path)) {dir.create(paste0("./RESULTS.", filename))}

outputDIR <- file.path(paste0("./RESULTS.", filename), as.character(Sys.Date()))

if (!dir.exists(outputDIR)) {dir.create(outputDIR)}

```

```{r, warning=FALSE, echo=FALSE, fig.height = 5, fig.width = 5, fig.align = "center}

j=4

filename <- "deltaFS_deltaLVmass"

param <- "FS"

DATA1 <- RawData[,c(1,3:4,6, which(colnames(RawData) == param))]

DATA1 <- DATA1 %>% pivot_wider(names_from = "echo", values_from = param)

DATA1$deltaLVEF <- DATA1$Echo1 - DATA1$Echo0

x1 <- 0

x2 <- 250

y1 <- -25

y2 <- 0

param2 <- "LVmass_corr"

DATA2 <- RawData[,c(1,3:4,6, which(colnames(RawData) == param2))]

DATA2 <- DATA2 %>% pivot_wider(names_from = "echo", values_from = param2)

DATA2$deltaLVmass <- (DATA2$Echo1 - DATA2$Echo0) /DATA2$Echo0 *100

DATA <- reduce(list(DATA1[,c("Animal_ID", "group", "deltaLVEF")], DATA2[,c("Animal_ID", "group", "deltaLVmass")]), left_join, by = c("Animal_ID","group"))

pmainm <- ggplot(DATA, aes_string(x = "deltaLVmass", y = "deltaLVEF", color = "group")) +

theme_tufte()+

theme(strip.text.x = element_text(face = "bold", size = 20),

panel.border = element_rect(linetype = "solid", fill = NA),

axis.title.x = element_text(face = "bold", size = 16),

axis.text.x = element_text(face = NULL, size = 12),

axis.title.y = element_text(face = "bold", size = 16, angle = 90),

axis.text.y = element_text(face = NULL, size = 12),

legend.title = element_blank()

) +

scale_color_manual(values = c("#66B2FF", "#3333FF", "#CC0066", "#990000"))+

geom_rect(aes(xmin = -Inf, xmax = 30, ymin = -Inf, ymax = Inf), size = 0.1, colour = "gray", fill = "gray", alpha = 0.03)+

geom_rect(aes(xmin = 30, xmax = Inf, ymin = -10, ymax = Inf), size = 0, colour = "gray", fill = "gray", alpha = 0.01)+

geom_point(size=2, alpha=0.5, width=0.25) +

geom_text_repel(aes(label = Animal_ID), size=2, show.legend = FALSE)+

annotate(geom = "text", label = "hypertrophy", x = Inf, y = Inf, hjust = 1.2, vjust = 1.5, size=4, family = "calibri")+

annotate(geom = "text", label = "heart failure", x = Inf, y = -Inf, hjust = 1.2, vjust = -1, size=4, family = "calibri")+

annotate(geom = "text", label = "healthy", x = -Inf, y = Inf, hjust = -0.2, vjust = 1.5, size=4, family = "calibri")+ #

xlim(x1,x2)+

ylim(y1,y2)+

geom_segment(aes(x = 30, xend = Inf, y = -10, yend = -10), linetype = "dotted", color = "black")+

geom_vline(xintercept = 30, linetype = "dotted", color = "black")+

xlab(expression(paste(Delta," LVmass [%]")))+

ylab(expression(paste(Delta," FS [%]")))+

theme(legend.position = "bottom")

DATA$group_material <- NA

for(i in 1:nrow(DATA)){

if(DATA$group[i] == "6-0 Prolene" | DATA$group[i] == "7-0 Prolene"){

DATA$group_material[i] <- "Prolene"

}else if(DATA$group[i] == "6-0 Silk" | DATA$group[i] == "7-0 Silk"){

DATA$group_material[i] <- "Silk"

}else {}

}

DATA$group_material <- as.factor(DATA$group_material)

DATA8 <- DATA[(DATA$group_material == levels(DATA$group_material)[1]),]; DATA8 <- DATA8 %>% drop_na("deltaLVmass")

DATA9 <- DATA[(DATA$group_material == levels(DATA$group_material)[2]),]; DATA9 <- DATA9 %>% drop_na("deltaLVmass")

if (shapiro.test(DATA8[["deltaLVmass"]])$p.value > 0.05 && shapiro.test(DATA9[["deltaLVmass"]])$p.value > 0.05){stat_test = "t.test"} else {stat_test = "wilcox.test"}

xplot <- ggplot(DATA, aes_string(x = "group_material", y = "deltaLVmass")) +

theme_tufte()+

theme(panel.border = element_rect(linetype = "solid", fill = NA),

axis.text.x = element_text(face = "bold", size = 18, angle = 45, hjust = 1),

) +

geom_boxplot(colour= "black", outlier.colour = NA, width = 0.5)+

stat_boxplot(colour= "black", geom ='errorbar', width = 0.3)+

geom_jitter(size=2, alpha=0.5, width=0.25, aes(color= group)) +

stat_compare_means(method = stat_test, aes(label = ..p.signif..), label.x = 1.5)+ #p.format

xlab("")+

ylab("")+

ylim(x1,x2)+

scale_color_manual(values = c("#66B2FF","#3333FF","#CC0066","#990000", "black"))+

coord_flip()

if (shapiro.test(DATA8[["deltaLVEF"]])$p.value > 0.05 && shapiro.test(DATA9[["deltaLVEF"]])$p.value > 0.05){stat_test = "t.test"} else {stat_test = "wilcox.test"}

yplot <- ggplot(DATA, aes_string(x = "group_material", y = "deltaLVEF")) +

theme_tufte()+

theme(panel.border = element_rect(linetype = "solid", fill = NA),

axis.text.x = element_text(face = "bold", size = 18, angle = 45, hjust = 1),

) +

geom_boxplot(colour= "black", outlier.colour = NA, width = 0.5)+ #lwd=0.2, alpha=0.5

stat_boxplot(colour= "black", geom ='errorbar', width = 0.3)+

geom_jitter(size=2, alpha=0.5, width=0.25, aes(color= group)) +

stat_compare_means(method = stat_test, aes(label = ..p.signif..), label.x = 1.5)+

xlab("")+

ylab("")+

ylim(y1,y2)+

scale_color_manual(values = c("#66B2FF","#3333FF","#CC0066","#990000", "black"))

# Combine the main plot and the density plot

PLOT <- insert_xaxis_grob(pmainm, xplot, grid::unit(3, "line"), position = "top")

PLOT <- insert_yaxis_grob(PLOT, yplot, grid::unit(3, "line"), position = "right")

ggsave(filename =paste0(j, ".", filename, ".png"), path = outputDIR, device = "png",

PLOT, width = 5, height = 5, dpi = 300, units = "in")

list_of_datasets <- list("Raw Data" = DATA)

write.xlsx(list_of_datasets, file = paste0(outputDIR, "/", i, ".",filename,".xlsx")) #save raw and summary data in excel

ggdraw(PLOT)

```

**SupplementaryCode-RepeatedMeasuresCorrelation.Rmd**

---

title: "The type of suture material affects transverse aortic constriction induced heart failure development in mice: A repeated measures correlation analysis"

author: "Benjamin Hackl"

date: "`r format(Sys.Date(), '%d.%m.%Y')`"

output:

pdf_document:

toc: false

number_sections: true

toc_depth: 2

df_print: kable

highlight: tango

latex_engine: xelatex

mainfont: "Calibri"

monofont: "Calibri Light"

---

```{r, include=FALSE, warning=FALSE}

rm(list=ls()) # clean your workspace

# list of required packages

.packages = c("here", "googlesheets4", "xlsx", "openxlsx", "readxl", "writexl", "tidyr", "plyr", "dplyr", "purrr", "ggplot2", "ggpubr", "ggpmisc", "ggrepel", "ggthemes", "ggpval", "ggforce", "cowplot", "lmerTest", "rstatix", "svglite","survminer", "survival", "RTCGA.clinical", "coxme", "gridExtra", "scales", "rmcorr", "rstudioapi", "pixiedust")

# install CRAN packages (if not already installed)

.inst <- .packages %in% installed.packages()

if(length(.packages[!.inst]) > 0) install.packages(.packages[!.inst])

# load packages into session

lapply(.packages, require, character.only=TRUE)

```

```{r, include=FALSE, warning=FALSE}

setwd(dirname(rstudioapi::getActiveDocumentContext()$path)) # set working directory to source file location

RawData <- read_excel("./RawData.xlsx", sheet= 2, na = "") # import raw data

RawData <- RawData %>% drop_na(Animal_ID)

RawData$group <- factor(RawData$group , levels=c("6-0 Silk", "7-0 Silk", "6-0 Prolene", "7-0 Prolene"))

RawData[,c(10,15:74)] <- sapply(RawData[,c(10,15:74)], as.numeric)

################################################################################

# convert dates of echo measurements to relative time

RawData$date_echo <- as.Date(RawData$date_echo, origin="1899-12-30")

RawData$date_surgery <- as.Date(RawData$date_surgery, origin="1899-12-30")

for (j in 1:nrow(RawData)) {

if(is.na(RawData$date_surgery[j])){

RawData$rel_time[j] <- NA

} else if(is.na(RawData$date_echo[j])){

RawData$rel_time[j] <- NA

} else{

RawData$rel_time[j] <- as.numeric(difftime(RawData$date_echo[j], RawData$date_surgery[j], units = "weeks"))

}

}

################################################################################

# define sub-analysis groups (material & size)

RmCorrData <- RawData

RmCorrData$group_material <- NA

for(i in 1:nrow(RmCorrData)){

if(RmCorrData$group[i] == "6-0 Prolene" | RmCorrData$group[i] == "7-0 Prolene"){

RmCorrData$group_material[i] <- "Prolene"

}else if(RmCorrData$group[i] == "6-0 Silk" | RmCorrData$group[i] == "7-0 Silk"){

RmCorrData$group_material[i] <- "Silk"

}else {}

}

RmCorrData$group_material <- factor(RmCorrData$group_material , levels=c("Silk", "Prolene"))

RmCorrData$group_size <- NA

for(i in 1:nrow(RmCorrData)){

if(RmCorrData$group[i] == "7-0 Silk" | RmCorrData$group[i] == "7-0 Prolene"){

RmCorrData$group_size[i] <- "7-0"

}else if(RmCorrData$group[i] == "6-0 Silk" | RmCorrData$group[i] == "6-0 Prolene"){

RmCorrData$group_size[i] <- "6-0"

}else {}

}

RmCorrData$group_size <- factor(RmCorrData$group_size , levels=c("6-0", "7-0"))

################################################################################

filename <- tools::file_path_sans_ext(basename(rstudioapi::getActiveDocumentContext()$path))

Ranalysis_path <- paste0("./RESULTS.", filename)

if (!dir.exists(Ranalysis_path)) {dir.create(paste0("./RESULTS.", filename))}

outputDIR <- file.path(paste0("./RESULTS.", filename), as.character(Sys.Date()))

if (!dir.exists(outputDIR)) {dir.create(outputDIR)}

################################################################################

### Repeated Measures Correlation setup, for details see Bakdash 2017 ##########

# new setup of "rmcorr" function ("isa"-function does not exist anymore, exchanged for "is.character")

rmcorr <- function(participant, measure1, measure2, dataset,

CI.level = 0.95,

CIs = c("analytic", "bootstrap"),

nreps = 100, bstrap.out = T) {

op <- options(contrasts = getOption("contrasts"))

on.exit(options(op))

options(contrasts = c("contr.sum", "contr.poly"))

args <- as.list(match.call())

Participant <- eval(args$participant, dataset, parent.frame())

if (is.character(Participant)&(length(Participant) == 1)){

Participant <- get(Participant, dataset)

}

Measure1 <- eval(args$measure1, dataset, parent.frame())

if (is.character(Participant)&(length(Measure1) == 1)){

Measure1 <- get(Measure1, dataset)

}

Measure2 <- eval(args$measure2, dataset, parent.frame())

if (is.character(Participant)&(length(Measure2) == 1)){

Measure2 <- get(Measure2, dataset)

}

if (!is.factor(Participant)){

Participant <- factor(Participant)

warning(paste("'", args$participant, "' coerced into a factor", sep = ""))

}

if (!is.numeric(Measure1) || !is.numeric(Measure2))

stop("'Measure 1' and 'Measure 2' must be numeric")

#check for missing values

newdat <- stats::na.omit(data.frame(Participant, Measure1, Measure2))

Participant <- newdat$Participant

Measure1 <- newdat$Measure1

Measure2 <- newdat$Measure2

CIs <- match.arg(CIs)

if (!is.numeric(CI.level) || CI.level <= 0 || CI.level >= 1){

stop("'CI.level' must be a numeric value between 0 and 1")

}

lmmodel <- stats::lm(Measure2 ~ Participant + Measure1)

lmslope <- stats::coef(lmmodel)["Measure1"]

errordf <- lmmodel$df.residual

# Direction of correlation based on whether slope is positive or negative

corrsign <- sign(lmslope)

# Drop each term for Type III sums of squares

type3rmcorr <- stats::drop1(lmmodel, ~., test="F" )

SSFactor <- type3rmcorr$'Sum of Sq'[3]

SSresidual <- type3rmcorr$RSS[1]

#correlation coefficient

rmcorrvalue <- as.numeric(corrsign * sqrt(SSFactor / (SSFactor + SSresidual)))

# Pvalue and confidence intervals

pvalue <- type3rmcorr$'Pr(>F)'[3]

#analytic

resamples <- NULL

if (CIs == "analytic"){

rmcorrvalueCI <- psych::r.con(rmcorrvalue, errordf, p = CI.level)

} else if (CIs == "bootstrap") {

nsubs <- length(levels(Participant))

if (!is.numeric(nreps)){stop("Specify the number of bootstrap resamples to take")}

cor.reps <- numeric(nreps)

for (i in 1:nreps){

split.by.sub <- split(newdat, newdat$Participant)

bs.df <- do.call(rbind,

lapply(split.by.sub,

function(x) x[sample(nrow(x), replace = T), ]))

bs.1 <- bs.df$Measure1

bs.2 <- bs.df$Measure2

bs.Part <- bs.df$Participant

repmodel<-stats::lm(bs.1 ~ bs.Part + bs.2)

repslope <- stats::coef(repmodel)["bs.2"]

errordf <- repmodel$df.residual

repsign <- sign(repslope)

type3rmcorr<-stats::drop1(repmodel, ~., test="F" )

SSFactor<-type3rmcorr$'Sum of Sq'[3]

SSresidual<-type3rmcorr$RSS[1]

cor.reps[i] <- as.numeric(repsign*sqrt(SSFactor/(SSFactor+SSresidual)))

}

CI.limits <- c((1-CI.level)/2, (1-CI.level)/2 + CI.level)

rmcorrvalueCI <- stats::quantile(cor.reps,probs=CI.limits)

resamples <- cor.reps

}

rmoutput <- list(r = rmcorrvalue, df = errordf, p = pvalue,

CI = rmcorrvalueCI,

CI.level = CI.level,

model = lmmodel,

vars = as.character(c(args$participant,args$measure1,args$measure2)))

if (bstrap.out) {rmoutput$resamples <- resamples}

class(rmoutput) <- "rmc"

return (rmoutput)

}

#' Print the results of a repeated measures correlation

#'

#' @param x An object of class "rmc", a result of a call to rmcorr.

#' @param ... additional arguments to \code{\link[base]{print}}.

#' @seealso \code{\link{rmcorr}}

#' @examples

#' ## Bland Altman 1995 data

#' blandrmc <- rmcorr(Subject, PacO2, pH, bland1995)

#' blandrmc

#' @export

print.rmc <- function(x, ...) {

cat("\nRepeated measures correlation\n\n")

cat("r\n")

cat(x$r)

cat("\n\ndegrees of freedom\n")

cat(x$df)

cat("\n\np-value\n")

cat(x$p)

cat("\n\n", x$CI.level*100, "% confidence interval\n", sep = "")

cat(x$CI,"\n\n")

}

################################################################################

### SETUP FOR GGPLOTS ##########################################################

annotate_npc <- function(label, x, y, ...){

ggplot2::annotation_custom(grid::textGrob(

x = unit(x, "npc"), y = unit(y, "npc"), label = label, ...))}

#########

theme_minimal = theme_bw() +

theme(

legend.position="none",

axis.line.x = element_line(color="black", size = 0.5),

axis.line.y = element_line(color="black", size = 0.5),

axis.text.x = element_text(size = 8),

axis.text.y = element_text(size = 8),

axis.title.x = element_text(size = 10),

axis.title.y = element_text(size = 10)

)

#########

compare.coeff <- function(b1,se1,b2,se2){

return((b1-b2)/sqrt(se1^2+se2^2))

}

#########

enough_data <- function(df, sex, group){

Data <- df[(df$sex == sex & df$treatment == group),] %>%

group_by(Animal_ID) %>% tally()

DataSub <- subset(Data, n > 2)

ifelse(nrow(DataSub) >= 3, TRUE, FALSE)

}

#########

enough_data_TAC <- function(df, sex, group){

Data <- df[(df$sex == sex & df$treatment == group),] %>%

group_by(Animal_ID) %>% tally()

DataSub <- subset(Data, n >= 2)

ifelse(nrow(DataSub) >4, TRUE, FALSE)

}

#########

not_enough_data <- function(df){

Data <- df %>% group_by(sex, treatment, echo) %>% tally()

ifelse(any(Data$n <= 3), TRUE, FALSE)

}

#########

makeStars <- function(x){

stars <- c("****", "***", "**", "*", "#", "ns")

vec <- c(0, 0.0001, 0.001, 0.01, 0.05, 0.1, 1)

i <- findInterval(x, vec)

stars[i]

}

#########

plot.rmc <- function(df, grouping, group, min_val, max_val){

if(grouping == "material"){

RmcData <- df[(df$group_material == group),]

}else if(grouping == "size"){

RmcData <- df[(df$group_size == group),]

}else if(grouping == "both"){

RmcData <- df[(df$group == group),]

}else{}

animal <- RmcData$Animal_ID

parameter <- RmcData[[param]]

time <- as.numeric(RmcData$rel_time)

rmcMatrix <-data.frame(cbind(animal, parameter, time))

rmcMatrix[, c(2:3)] <- sapply(rmcMatrix[, c(2:3)], as.numeric)

rmcMatrix[, 1] <- sapply(rmcMatrix[, 1], as.factor)

rmcData <- rmcMatrix %>% drop_na(parameter)

model.rmc <- rmcorr(participant = animal, measure1 = time, measure2 = parameter, dataset = rmcData)

animalmeanx <- aggregate(rmcMatrix$parameter, by = list(rmcMatrix$animal), mean)

animalmeany <- aggregate(rmcMatrix$time, by = list(rmcMatrix$animal), mean)

rmc.r <- sprintf("%.2f", round(model.rmc$r, 2))

rmc.p <- pval_string(model.rmc$p)

rmc.slope <- round(model.rmc$model$coefficients[length(model.rmc$model$coefficients)], 3)

slope <<- summary(model.rmc$model)$coefficients[length(model.rmc$model$coefficients),1]

se <<- summary(model.rmc$model)$coefficients[length(model.rmc$model$coefficients),2]

title = paste0(group)

ggplot(rmcData, aes(x = time, y = parameter, group = animal, color = animal)) +

geom_point(aes(colour = animal)) +

geom_line(aes(y = model.rmc$model$fitted.values), linetype = 1) +

theme_minimal +

labs(title = title,

x = "time post TAC [weeks]",

y = axistitle) +

#scale_colour_brewer(palette = "BrBG") +

theme(plot.title = element_text(hjust = 0.5)) +

geom_line(linetype = 3) +

ylim(min_val,max_val)+

annotate_npc(x = 0.3, y = 0.9, label = bquote(italic(r[rm]) ~ "=" ~ .(rmc.r)))+

annotate_npc(x = 0.3, y = 0.75, label = bquote(italic('p') ~ .(rmc.p)))+

annotate_npc(x = 0.7, y = 0.1, label = bquote(italic('slope') ~ "=" ~ .(rmc.slope)))

}

plot.stats <- function(slope.a, se.a, slope.b, se.b, slope.c, se.c, slope.d, se.d){

p_value1 = 2*pnorm(-abs(compare.coeff(slope.a,se.a,slope.b,se.b)))

p_value2 = 2*pnorm(-abs(compare.coeff(slope.a,se.a,slope.c,se.c)))

p_value3 = 2*pnorm(-abs(compare.coeff(slope.a,se.a,slope.d,se.d)))

p_value4 = 2*pnorm(-abs(compare.coeff(slope.b,se.b,slope.c,se.c)))

p_value5 = 2*pnorm(-abs(compare.coeff(slope.b,se.b,slope.d,se.d)))

p_value6 = 2*pnorm(-abs(compare.coeff(slope.c,se.c,slope.d,se.d)))

rmcorr_table1 <- data.frame(c("6-0 Silk", "6-0 Silk", "6-0 Silk", "7-0 Silk", "7-0 Silk", "6-0 Prolene"),

c(round(slope.a,2),round(slope.a,2),round(slope.a,2), round(slope.b,2),round(slope.b,2),round(slope.c,2)),

c(round(se.a,2),round(se.a,2),round(se.a,2), round(se.b,2),round(se.b,2),round(se.c,2)),

c("7-0 Silk", "6-0 Prolene", "7-0 Prolene", "6-0 Prolene", "7-0 Prolene", "7-0 Prolene"),

c(round(slope.b,2),round(slope.c,2),round(slope.d,2), round(slope.c,2),round(slope.d,2),round(slope.d,2)),

c(round(se.b,2),round(se.c,2),round(se.d,2), round(se.c,2),round(se.d,2),round(se.d,2)),

c(round(p_value1,3),round(p_value2,3),round(p_value3,3),round(p_value4,3),round(p_value5,3),round(p_value6,3)),

c(makeStars(p_value1),makeStars(p_value2),makeStars(p_value3),makeStars(p_value4),makeStars(p_value5),makeStars(p_value6))

)

names(rmcorr_table1) = c("group1","slope","sem","group2","slope", "sem","p value", "signif.")

print(rmcorr_table1)

}

################################################################################

plot.stats2 <- function(slope.a, se.a, slope.b, se.b, slope.c, se.c, slope.d, se.d){

p_value1 = 2*pnorm(-abs(compare.coeff(slope.a,se.a,slope.b,se.b)))

p_value2 = 2*pnorm(-abs(compare.coeff(slope.c,se.c,slope.d,se.d)))

rmcorr_table1 <- data.frame(c("Silk", "6-0"),

c(round(slope.a,2),round(slope.c,2)),

c(round(se.a,2),round(se.c,2)),

c("Prolene", "7-0"),

c(round(slope.b,2),round(slope.d,2)),

c(round(se.b,2),round(se.d,2)),

c(round(p_value1,3),round(p_value2,3)),

c(makeStars(p_value1),makeStars(p_value2))

)

names(rmcorr_table1) = c("group1","slope","sem","group2","slope", "sem","p value", "signif.")

print(rmcorr_table1)

}

################################################################################

################################################################################

plot.classic <- function(df){

DATA <- df

DATA <- DATA %>% drop_na(param)

STAT <- DATA %>% group_by(group, echo) %>% dplyr::summarise_at(vars(paste0(param)), funs(mean, sd, n= length))

plot0 <- ggplot(DATA, aes_string(x = "group", y = param, color = "group")) +

theme_tufte()+

theme(legend.text = element_text(size=16),

panel.border = element_rect(linetype = "solid", fill = NA),

strip.text = element_text(face = "bold",size = 16),

axis.text.x = element_text(face = "bold", size = 18, angle = 45, hjust = 1),

axis.title.y = element_text(face = "bold", size = 20, angle = 90),

axis.text.y = element_text(face = NULL, size = 16)

) +

geom_boxplot(colour= "black", outlier.colour = NA, width = 0.5)+ #lwd=0.2, alpha=0.5

stat_boxplot(colour= "black", geom ='errorbar', width = 0.3)+

geom_jitter(size=3, alpha=0.5, width=0.25) +

scale_color_manual(values = c("#CC0066","#66B2FF","#990000","#3333FF","black"))+ #palette =

stat_compare_means(method = "anova", label = "p.format")+ #, aes(label = ..p.signif..), label.x = 1.5)+# label="p.signif", tip.length=0)+

geom_text(data = STAT, size=3.5, aes(y = 0, label = n, family = "calibri", color = "black"))+

#annotate(geom = "text", label = stat_test, x = -Inf, y = Inf, hjust = -0.1, vjust = 1, size=4, family = "calibri",)+

ylab(axistitle)+

xlab("")+

facet_wrap( ~ echo, ncol = 4)+

theme(legend.position = "none")

ggsave(filename =paste0("Anova_all_", param, ".png"), path = outputDIR, device = "png",

plot0, width = 6, height = 5, dpi = 300, units = "in")

plot(ggdraw(plot0))

echos <- c("Echo0", "Echo1", "Echo3")

for (k in 1:length(echos)){

DATA <- RmCorrData[(RmCorrData$echo == echos[k]),]

DATA <- DATA %>% drop_na(param)

STAT <- DATA %>% group_by(group_material) %>% dplyr::summarise_at(vars(paste0(param)), funs(mean, sd, n= length))

DATA1 <- DATA[(DATA$group == levels(DATA$group)[1]),]; DATA1 <- DATA1 %>% drop_na(param)

DATA2 <- DATA[(DATA$group == levels(DATA$group)[2]),]; DATA2 <- DATA2 %>% drop_na(param)

if (shapiro.test(DATA1[[param]])$p.value > 0.05){stat_test = "t.test"} else {stat_test = "wilcox.test"}

plot1 <- ggplot(DATA, aes_string(x = "group_material", y = param, color = "group_material")) +

theme_tufte()+

theme(legend.text = element_text(size=16),

panel.border = element_rect(linetype = "solid", fill = NA),

axis.text.x = element_text(face = "bold", size = 18),

axis.title.y = element_text(face = "bold", size = 20, angle = 90),

axis.text.y = element_text(face = NULL, size = 16)

) +

geom_boxplot(colour= "black", outlier.colour = NA, width = 0.5)+ #lwd=0.2, alpha=0.5

stat_boxplot(colour= "black", geom ='errorbar', width = 0.3)+

geom_jitter(size=5, alpha=0.5, width=0.25) +

scale_color_manual(values = c("black","#990000","#3333FF"))+ #palette =

stat_compare_means(method = stat_test, aes(label = ..p.signif..), label.x = 1.5)+# label="p.signif", tip.length=0)+

geom_text(data = STAT, size=3.5, aes(y = 0, label = paste0("n = ", n), family = "calibri", color = "black"))+

annotate(geom = "text", label = stat_test, x = -Inf, y = Inf, hjust = -0.1, vjust = 1, size=4, family = "calibri",)+

ylab(axistitle)+

xlab("")+

theme(legend.position = "none")

STAT <- DATA %>% group_by(group_size) %>% dplyr::summarise_at(vars(paste0(param)), funs(mean, sd, n= length))

DATA3 <- DATA[(DATA$group_size == levels(DATA$group_size)[1]),]; DATA3 <- DATA3 %>% drop_na(param)

DATA4 <- DATA[(DATA$group_size == levels(DATA$group_size)[2]),]; DATA4 <- DATA4 %>% drop_na(param)

if (shapiro.test(DATA3[[param]])$p.value > 0.05){stat_test = "t.test"} else {stat_test = "wilcox.test"}

plot2 <- ggplot(DATA, aes_string(x = "group_size", y = param, color = "group_size")) +

theme_tufte()+

theme(legend.text = element_text(size=16),

panel.border = element_rect(linetype = "solid", fill = NA),

axis.text.x = element_text(face = "bold", size = 18),

axis.title.y = element_text(face = "bold", size = 20, angle = 90),

axis.text.y = element_text(face = NULL, size = 16)

) +

geom_boxplot(colour= "black", outlier.colour = NA, width = 0.5)+ #lwd=0.2, alpha=0.5

stat_boxplot(colour= "black", geom ='errorbar', width = 0.3)+

geom_jitter(size=5, alpha=0.5, width=0.25) +

scale_color_manual(values = c("grey","black","black"))+ #palette =

stat_compare_means(method = stat_test, aes(label = ..p.signif..), label.x = 1.5)+# label="p.signif", tip.length=0)+

geom_text(data = STAT, size=3.5, aes(y = 0, label = paste0("n = ", n), family = "calibri", color = "black"))+

annotate(geom = "text", label = stat_test, x = -Inf, y = Inf, hjust = -0.1, vjust = 1, size=4, family = "calibri",)+

ylab(axistitle)+

xlab("")+

theme(legend.position = "none")

PLOT2 <<- ggarrange(plot1, plot2,

labels = c("A", "B"),

ncol = 2, nrow = 1)

ggsave(filename =paste0("Anova_grouped_", param, "_",echos[k], ".png"), path = outputDIR, device = "png",

PLOT2, width = 5, height = 5, dpi = 300, units = "in")

plot(ggdraw(PLOT2))

}

}

```

```{r}

param = "BW"

axistitle <- expression(paste("body weight [g]"))

DATA <- RmCorrData

################################################################################

plot.a <- plot.rmc(DATA, "both", '6-0 Silk', 0, 40); slope.a = slope; se.a = se

plot.b <- plot.rmc(DATA, "both", '7-0 Silk', 0, 40); slope.b = slope; se.b = se

plot.c <- plot.rmc(DATA, "both", '6-0 Prolene', 0, 40); slope.c = slope; se.c = se

plot.d <- plot.rmc(DATA, "both", '7-0 Prolene', 0, 40); slope.d = slope; se.d = se

stats <- ggtexttable(plot.stats(slope.a, se.a, slope.b, se.b, slope.c, se.c, slope.d, se.d),

rows = NULL, theme = ttheme("light"))

PLOT1 <- ggarrange(ggarrange(plot.a, plot.b, plot.c, plot.d,

labels = c("A", "B", "C", "D"),

nrow = 2, ncol = 2, common.legend = FALSE),

ggarrange(stats, labels = "E"),

nrow = 2, heights = c(2:1))

ggsave(filename =paste0("RmCorr_all_", param, ".png"), path = outputDIR, device = "png",

PLOT1, width = 5.5, height = 7.5, dpi = 300, units = "in")

print(PLOT1)

################################################################################

plot.e <- plot.rmc(DATA, "material", 'Silk', 0, 40); slope.e = slope; se.e = se

plot.f <- plot.rmc(DATA, 'material', 'Prolene', 0, 40); slope.f = slope; se.f = se

plot.g <- plot.rmc(DATA, 'size', '6-0', 0, 40); slope.g = slope; se.g = se

plot.h <- plot.rmc(DATA, 'size', '7-0', 0, 40); slope.h = slope; se.h = se

stats <- ggtexttable(plot.stats2(slope.e, se.e, slope.f, se.f, slope.g, se.g, slope.h, se.h),

rows = NULL, theme = ttheme("light"))

PLOT2 <- ggarrange(ggarrange(plot.e, plot.f, plot.g, plot.h,

labels = c("A", "B", "C", "D"),

nrow = 2, ncol = 2, common.legend = FALSE),

ggarrange(stats, labels = "E"),

nrow = 2, heights = c(3,0.75))

ggsave(filename =paste0("RmCorr_", param, ".png"), path = outputDIR, device = "png",

PLOT2, width = 5.5, height = 7.5, dpi = 300, units = "in")

write.xlsx(DATA, file = paste0(outputDIR, "/", "RmCorr_",param,".xlsx"))

print(PLOT2)

################################################################################

plot.classic(RmCorrData)

```

```{r}

param = "LVEF"

axistitle <- expression(paste("LV ejection fraction [%]"))

DATA <- RmCorrData

################################################################################

plot.a <- plot.rmc(DATA, "both", '6-0 Silk', 0, 65); slope.a = slope; se.a = se

plot.b <- plot.rmc(DATA, "both", '7-0 Silk', 0, 65); slope.b = slope; se.b = se

plot.c <- plot.rmc(DATA, "both", '6-0 Prolene', 0, 65); slope.c = slope; se.c = se

plot.d <- plot.rmc(DATA, "both", '7-0 Prolene', 0, 65); slope.d = slope; se.d = se

stats <- ggtexttable(plot.stats(slope.a, se.a, slope.b, se.b, slope.c, se.c, slope.d, se.d),

rows = NULL, theme = ttheme("light"))

PLOT1 <- ggarrange(ggarrange(plot.a, plot.b, plot.c, plot.d,

labels = c("A", "B", "C", "D"),

nrow = 2, ncol = 2, common.legend = FALSE),

ggarrange(stats, labels = "E"),

nrow = 2, heights = c(2:1))

ggsave(filename =paste0("RmCorr_all_", param, ".png"), path = outputDIR, device = "png",

PLOT1, width = 5.5, height = 7.5, dpi = 300, units = "in")

print(PLOT1)

################################################################################

plot.e <- plot.rmc(DATA, "material", 'Silk', 0, 65); slope.e = slope; se.e = se

plot.f <- plot.rmc(DATA, 'material', 'Prolene', 0, 65); slope.f = slope; se.f = se

plot.g <- plot.rmc(DATA, 'size', '6-0', 0, 65); slope.g = slope; se.g = se

plot.h <- plot.rmc(DATA, 'size', '7-0', 0, 65); slope.h = slope; se.h = se

stats <- ggtexttable(plot.stats2(slope.e, se.e, slope.f, se.f, slope.g, se.g, slope.h, se.h),

rows = NULL, theme = ttheme("light"))

PLOT2 <- ggarrange(ggarrange(plot.e, plot.f, plot.g, plot.h,

labels = c("A", "B", "C", "D"),

nrow = 2, ncol = 2, common.legend = FALSE),

ggarrange(stats, labels = "E"),

nrow = 2, heights = c(3,0.75))

ggsave(filename =paste0("RmCorr_", param, ".png"), path = outputDIR, device = "png",

PLOT2, width = 5.5, height = 7.5, dpi = 300, units = "in")

write.xlsx(DATA, file = paste0(outputDIR, "/", "RmCorr_",param,".xlsx"))

print(PLOT2)

################################################################################

plot.classic(RmCorrData)

```

```{r}

param = "LVmass_corr"

axistitle <- expression(paste("left ventricular mass [mg]"))

DATA <- RmCorrData

################################################################################

plot.a <- plot.rmc(DATA, "both", '6-0 Silk', 0, 300); slope.a = slope; se.a = se

plot.b <- plot.rmc(DATA, "both", '7-0 Silk', 0, 300); slope.b = slope; se.b = se

plot.c <- plot.rmc(DATA, "both", '6-0 Prolene', 0, 300); slope.c = slope; se.c = se

plot.d <- plot.rmc(DATA, "both", '7-0 Prolene', 0, 300); slope.d = slope; se.d = se

stats <- ggtexttable(plot.stats(slope.a, se.a, slope.b, se.b, slope.c, se.c, slope.d, se.d),

rows = NULL, theme = ttheme("light"))

PLOT1 <- ggarrange(ggarrange(plot.a, plot.b, plot.c, plot.d,

labels = c("A", "B", "C", "D"),

nrow = 2, ncol = 2, common.legend = FALSE),

ggarrange(stats, labels = "E"),

nrow = 2, heights = c(2:1))

ggsave(filename =paste0("RmCorr_all_", param, ".png"), path = outputDIR, device = "png",

PLOT1, width = 5.5, height = 7.5, dpi = 300, units = "in")

print(PLOT1)

################################################################################

plot.e <- plot.rmc(DATA, "material", 'Silk', 0, 300); slope.e = slope; se.e = se

plot.f <- plot.rmc(DATA, 'material', 'Prolene', 0, 300); slope.f = slope; se.f = se

plot.g <- plot.rmc(DATA, 'size', '6-0', 0, 300); slope.g = slope; se.g = se

plot.h <- plot.rmc(DATA, 'size', '7-0', 0, 300); slope.h = slope; se.h = se

stats <- ggtexttable(plot.stats2(slope.e, se.e, slope.f, se.f, slope.g, se.g, slope.h, se.h),

rows = NULL, theme = ttheme("light"))

PLOT2 <- ggarrange(ggarrange(plot.e, plot.f, plot.g, plot.h,

labels = c("A", "B", "C", "D"),

nrow = 2, ncol = 2, common.legend = FALSE),

ggarrange(stats, labels = "E"),

nrow = 2, heights = c(3,0.75))

ggsave(filename =paste0("RmCorr_", param, ".png"), path = outputDIR, device = "png",

PLOT2, width = 5.5, height = 7.5, dpi = 300, units = "in")

write.xlsx(DATA, file = paste0(outputDIR, "/", "RmCorr_",param,".xlsx"))

print(PLOT2)

################################################################################

plot.classic(RmCorrData)

```

```{r}

param = "HW_BW"

axistitle <- expression(paste("LVmass to BW ratio [mg/g]"))

DATA <- RmCorrData

min <- 0

max <- 12

################################################################################

plot.a <- plot.rmc(DATA, "both", '6-0 Silk', min, max); slope.a = slope; se.a = se

plot.b <- plot.rmc(DATA, "both", '7-0 Silk', min, max); slope.b = slope; se.b = se

plot.c <- plot.rmc(DATA, "both", '6-0 Prolene', min, max); slope.c = slope; se.c = se

plot.d <- plot.rmc(DATA, "both", '7-0 Prolene', min, max); slope.d = slope; se.d = se

stats <- ggtexttable(plot.stats(slope.a, se.a, slope.b, se.b, slope.c, se.c, slope.d, se.d),

rows = NULL, theme = ttheme("light"))

PLOT1 <- ggarrange(ggarrange(plot.a, plot.b, plot.c, plot.d,

labels = c("A", "B", "C", "D"),

nrow = 2, ncol = 2, common.legend = FALSE),

ggarrange(stats, labels = "E"),

nrow = 2, heights = c(2:1))

ggsave(filename =paste0("RmCorr_all_", param, ".png"), path = outputDIR, device = "png",

PLOT1, width = 5.5, height = 7.5, dpi = 300, units = "in")

print(PLOT1)

################################################################################

plot.e <- plot.rmc(DATA, "material", 'Silk', min, max); slope.e = slope; se.e = se

plot.f <- plot.rmc(DATA, 'material', 'Prolene', min, max); slope.f = slope; se.f = se

plot.g <- plot.rmc(DATA, 'size', '6-0', min, max); slope.g = slope; se.g = se

plot.h <- plot.rmc(DATA, 'size', '7-0', min, max); slope.h = slope; se.h = se

stats <- ggtexttable(plot.stats2(slope.e, se.e, slope.f, se.f, slope.g, se.g, slope.h, se.h),

rows = NULL, theme = ttheme("light"))

PLOT2 <- ggarrange(ggarrange(plot.e, plot.f, plot.g, plot.h,

labels = c("A", "B", "C", "D"),

nrow = 2, ncol = 2, common.legend = FALSE),

ggarrange(stats, labels = "E"),

nrow = 2, heights = c(3,0.75))

ggsave(filename =paste0("RmCorr_", param, ".png"), path = outputDIR, device = "png",

PLOT2, width = 5.5, height = 7.5, dpi = 300, units = "in")

write.xlsx(DATA, file = paste0(outputDIR, "/", "RmCorr_",param,".xlsx"))

print(PLOT2)

################################################################################

plot.classic(RmCorrData)

```

```{r}

param = "CO"

axistitle <- expression(paste("cardiac output [mL/min]"))

DATA <- RmCorrData

################################################################################

plot.a <- plot.rmc(DATA, "both", '6-0 Silk', 0, 25); slope.a = slope; se.a = se

plot.b <- plot.rmc(DATA, "both", '7-0 Silk', 0, 25); slope.b = slope; se.b = se

plot.c <- plot.rmc(DATA, "both", '6-0 Prolene', 0, 25); slope.c = slope; se.c = se

plot.d <- plot.rmc(DATA, "both", '7-0 Prolene', 0, 25); slope.d = slope; se.d = se

stats <- ggtexttable(plot.stats(slope.a, se.a, slope.b, se.b, slope.c, se.c, slope.d, se.d),

rows = NULL, theme = ttheme("light"))

PLOT1 <- ggarrange(ggarrange(plot.a, plot.b, plot.c, plot.d,

labels = c("A", "B", "C", "D"),

nrow = 2, ncol = 2, common.legend = FALSE),

ggarrange(stats, labels = "E"),

nrow = 2, heights = c(2:1))

ggsave(filename =paste0("RmCorr_all_", param, ".png"), path = outputDIR, device = "png",

PLOT1, width = 5.5, height = 7.5, dpi = 300, units = "in")

print(PLOT1)

################################################################################

plot.e <- plot.rmc(DATA, "material", 'Silk', 0, 25); slope.e = slope; se.e = se

plot.f <- plot.rmc(DATA, 'material', 'Prolene', 0, 25); slope.f = slope; se.f = se

plot.g <- plot.rmc(DATA, 'size', '6-0', 0, 25); slope.g = slope; se.g = se

plot.h <- plot.rmc(DATA, 'size', '7-0', 0, 25); slope.h = slope; se.h = se

stats <- ggtexttable(plot.stats2(slope.e, se.e, slope.f, se.f, slope.g, se.g, slope.h, se.h),

rows = NULL, theme = ttheme("light"))

PLOT2 <- ggarrange(ggarrange(plot.e, plot.f, plot.g, plot.h,

labels = c("A", "B", "C", "D"),

nrow = 2, ncol = 2, common.legend = FALSE),

ggarrange(stats, labels = "E"),

nrow = 2, heights = c(3,0.75))

ggsave(filename =paste0("RmCorr_", param, ".png"), path = outputDIR, device = "png",

PLOT2, width = 5.5, height = 7.5, dpi = 300, units = "in")

write.xlsx(DATA, file = paste0(outputDIR, "/", "RmCorr_",param,".xlsx"))

print(PLOT2)

################################################################################

plot.classic(RmCorrData)

```

```{r}

param = "SV"

axistitle <- expression(paste("stroke volume [µL]"))

DATA <- RmCorrData

################################################################################

plot.a <- plot.rmc(DATA, "both", '6-0 Silk', 0, 60); slope.a = slope; se.a = se

plot.b <- plot.rmc(DATA, "both", '7-0 Silk', 0, 60); slope.b = slope; se.b = se

plot.c <- plot.rmc(DATA, "both", '6-0 Prolene', 0, 60); slope.c = slope; se.c = se

plot.d <- plot.rmc(DATA, "both", '7-0 Prolene', 0, 60); slope.d = slope; se.d = se

stats <- ggtexttable(plot.stats(slope.a, se.a, slope.b, se.b, slope.c, se.c, slope.d, se.d),

rows = NULL, theme = ttheme("light"))

PLOT1 <- ggarrange(ggarrange(plot.a, plot.b, plot.c, plot.d,

labels = c("A", "B", "C", "D"),

nrow = 2, ncol = 2, common.legend = FALSE),

ggarrange(stats, labels = "E"),

nrow = 2, heights = c(2:1))

ggsave(filename =paste0("RmCorr_all_", param, ".png"), path = outputDIR, device = "png",

PLOT1, width = 5.5, height = 7.5, dpi = 300, units = "in")

print(PLOT1)

################################################################################

plot.e <- plot.rmc(DATA, "material", 'Silk', 0, 60); slope.e = slope; se.e = se

plot.f <- plot.rmc(DATA, 'material', 'Prolene', 0, 60); slope.f = slope; se.f = se

plot.g <- plot.rmc(DATA, 'size', '6-0', 0, 60); slope.g = slope; se.g = se

plot.h <- plot.rmc(DATA, 'size', '7-0', 0, 60); slope.h = slope; se.h = se

stats <- ggtexttable(plot.stats2(slope.e, se.e, slope.f, se.f, slope.g, se.g, slope.h, se.h),

rows = NULL, theme = ttheme("light"))

PLOT2 <- ggarrange(ggarrange(plot.e, plot.f, plot.g, plot.h,

labels = c("A", "B", "C", "D"),

nrow = 2, ncol = 2, common.legend = FALSE),

ggarrange(stats, labels = "E"),

nrow = 2, heights = c(3,0.75))

ggsave(filename =paste0("RmCorr_", param, ".png"), path = outputDIR, device = "png",

PLOT2, width = 5.5, height = 7.5, dpi = 300, units = "in")

write.xlsx(DATA, file = paste0(outputDIR, "/", "RmCorr_",param,".xlsx"))

print(PLOT2)

################################################################################

plot.classic(RmCorrData)

```

```{r}

param = "pgrad"

DATA <- RmCorrData

DATA[, param] <- sapply(DATA[, param], as.numeric)

plot.rmc.pgrad <- function(df, grouping, group, min_val, max_val){

if(grouping == "material"){

RmcData <- df[(df$group_material == group),]

}else if(grouping == "size"){

RmcData <- df[(df$group_size == group),]

}else if(grouping == "both"){

RmcData <- df[(df$group == group),]

}else{}

animal <- RmcData$Animal_ID

parameter <- RmcData[[param]]

time <- as.numeric(RmcData$rel_time)

rmcMatrix <-data.frame(cbind(animal, parameter, time))

rmcMatrix[, c(2:3)] <- sapply(rmcMatrix[, c(2:3)], as.numeric)

rmcMatrix[, 1] <- sapply(rmcMatrix[, 1], as.factor)

rmcData <- rmcMatrix %>% drop_na(parameter)

rmcData2 <- rmcData[(rmcData$time >= 0.1),]

model.rmc <- rmcorr(participant = animal, measure1 = time, measure2 = parameter, dataset = rmcData2)

animalmeanx <- aggregate(rmcMatrix$parameter, by = list(rmcMatrix$animal), mean)

animalmeany <- aggregate(rmcMatrix$time, by = list(rmcMatrix$animal), mean)

rmc.r <- sprintf("%.2f", round(model.rmc$r, 2))

rmc.p <- pval_string(model.rmc$p)

rmc.slope <- round(model.rmc$model$coefficients[length(model.rmc$model$coefficients)], 3)

slope <<- summary(model.rmc$model)$coefficients[length(model.rmc$model$coefficients),1]

se <<- summary(model.rmc$model)$coefficients[length(model.rmc$model$coefficients),2]

title = paste0(group)

ggplot() +

geom_point(data = rmcData, aes(x = time, y = parameter, group = animal, colour = animal)) +

geom_line(data = rmcData2, aes(x = time, y = model.rmc$model$fitted.values, group = animal, color = animal), linetype = 1) +

theme_minimal +

labs(title = title,

x = "time post TAC [weeks]",

y = axistitle) +

#scale_colour_brewer(palette = "BrBG") +

theme(plot.title = element_text(hjust = 0.5)) +

geom_line(data = rmcData, aes(x = time, y = parameter, group = animal, colour = animal), linetype = 3) +

ylim(min_val,max_val)+

annotate_npc(x = 0.3, y = 0.9, label = bquote(italic(r[rm]) ~ "=" ~ .(rmc.r)))+

annotate_npc(x = 0.3, y = 0.75, label = bquote(italic('p') ~ .(rmc.p)))+

annotate_npc(x = 0.7, y = 0.1, label = bquote(italic('slope') ~ "=" ~ .(rmc.slope)))

}

axistitle <- expression(paste(Delta, "p [mmHg]"))

#plot.a <- plot.rmc.pgrad(DATA, "both", '6-0 Silk', 0, 100); slope.a = slope; se.a = se

#plot.b <- plot.rmc.pgrad(DATA, 'both', '7-0 Silk', 0, 100); slope.b = slope; se.b = se

#plot.c <- plot.rmc.pgrad(DATA, 'both', '6-0 Prolene', 0, 100); slope.c = slope; se.c = se

#plot.d <- plot.rmc.pgrad(DATA, 'both', '7-0 Prolene', 0, 100); slope.d = slope; se.d = se

plot.e <- plot.rmc.pgrad(DATA, "material", 'Silk', 0, 100); slope.e = slope; se.e = se

plot.f <- plot.rmc.pgrad(DATA, 'material', 'Prolene', 0, 100); slope.f = slope; se.f = se

plot.g <- plot.rmc.pgrad(DATA, 'size', '6-0', 0, 100); slope.g = slope; se.g = se

plot.h <- plot.rmc.pgrad(DATA, 'size', '7-0', 0, 100); slope.h = slope; se.h = se

stats <- ggtexttable(plot.stats2(slope.e, se.e, slope.f, se.f, slope.g, se.g, slope.h, se.h),

rows = NULL, theme = ttheme("light"))

PLOT2 <- ggarrange(ggarrange(plot.e, plot.f, plot.g, plot.h,

labels = c("A", "B", "C", "D"),

nrow = 2, ncol = 2, common.legend = FALSE),

ggarrange(stats, labels = "E"),

nrow = 2, heights = c(3,0.75))

ggsave(filename =paste0("RmCorr_", param, ".png"), path = outputDIR, device = "png",

PLOT2, width = 5.5, height = 7.5, dpi = 300, units = "in")

write.xlsx(DATA, file = paste0(outputDIR, "/", "RmCorr_",param,".xlsx"))

print(PLOT2)

DATA <- DATA %>% drop_na(param)

STAT <- DATA %>% group_by(group, echo) %>% dplyr::summarise_at(vars(paste0(param)), funs(mean, sd, n= length))

plot0 <- ggplot(DATA, aes_string(x = "group", y = param, color = "group")) +

theme_tufte()+

theme(legend.text = element_text(size=16),

panel.border = element_rect(linetype = "solid", fill = NA),

strip.text = element_text(face = "bold",size = 16),

axis.text.x = element_text(face = "bold", size = 18, angle = 45, hjust = 1),

axis.title.y = element_text(face = "bold", size = 20, angle = 90),

axis.text.y = element_text(face = NULL, size = 16)

) +

geom_boxplot(colour= "black", outlier.colour = NA, width = 0.5)+ #lwd=0.2, alpha=0.5

stat_boxplot(colour= "black", geom ='errorbar', width = 0.3)+

geom_jitter(size=3, alpha=0.5, width=0.25) +

scale_color_manual(values = c("#CC0066","#66B2FF","#990000","#3333FF","black"))+ #palette =

stat_compare_means(method = "anova", label = "p.format")+ #, aes(label = ..p.signif..), label.x = 1.5)+# label="p.signif", tip.length=0)+

geom_text(data = STAT, size=3.5, aes(y = 0, label = n, family = "calibri", color = "black"))+

#annotate(geom = "text", label = stat_test, x = -Inf, y = Inf, hjust = -0.1, vjust = 1, size=4, family = "calibri",)+

ylab(axistitle)+

xlab("")+

facet_wrap( ~ echo, ncol = 4)+

theme(legend.position = "none")

ggsave(filename =paste0("Anova_all_", param, ".png"), path = outputDIR, device = "png",

plot0, width = 6, height = 5, dpi = 300, units = "in")

plot(ggdraw(plot0))

```

```{r}

param = "Temp"

axistitle <- expression(paste("body temperature [°C]"))

DATA <- RmCorrData

################################################################################

DATA <- DATA %>% drop_na(param)

STAT <- DATA %>% group_by(group_material, echo) %>% dplyr::summarise_at(vars(paste0(param)), funs(mean, sd, n= length))

plot0 <- ggplot(DATA, aes_string(x = "group_material", y = param)) +

theme_tufte()+

theme(legend.text = element_text(size=16),

panel.border = element_rect(linetype = "solid", fill = NA),

strip.text = element_text(face = "bold",size = 16),

axis.text.x = element_text(face = "bold", size = 18, angle = 45, hjust = 1),

axis.title.y = element_text(face = "bold", size = 20, angle = 90),

axis.text.y = element_text(face = NULL, size = 16)

) +

geom_boxplot(colour= "black", outlier.colour = NA, width = 0.5)+ #lwd=0.2, alpha=0.5

stat_boxplot(colour= "black", geom ='errorbar', width = 0.3)+

geom_jitter(size=3, alpha=0.5, width=0.25, aes(color= group)) +

scale_color_manual(values = c("#CC0066","#66B2FF","#990000","#3333FF","black"))+ #palette =

stat_compare_means(method = "anova", label = "p.format")+ #, aes(label = ..p.signif..), label.x = 1.5)+# label="p.signif", tip.length=0)+

geom_text(data = STAT, size=3.5, aes(y = 0, label = n, family = "calibri", color = "black"))+

#annotate(geom = "text", label = stat_test, x = -Inf, y = Inf, hjust = -0.1, vjust = 1, size=4, family = "calibri",)+

ylab(axistitle)+

xlab("")+

ylim(35.5,38.5)+

facet_wrap( ~ echo, ncol = 4)+

theme(legend.position = "none")

ggsave(filename =paste0("Anova_all_", param, ".png"), path = outputDIR, device = "png",

plot0, width = 6, height = 5, dpi = 300, units = "in")

plot(ggdraw(plot0))

```

**SupplementaryCode-SuitureSurvival.Rmd**

---

title: "The type of suture material affects transverse aortic constriction induced heart failure development in mice: A repeated measures correlation analysis"

author: "Benjamin Hackl"

date: "`r format(Sys.Date(), '%d.%m.%Y')`"

output:

pdf_document:

toc: false

number_sections: true

toc_depth: 2

df_print: kable

highlight: tango

latex_engine: xelatex

mainfont: "Calibri"

monofont: "Calibri Light"

---

```{r, include=FALSE, warning=FALSE}

rm(list=ls()) # clean your workspace

# list of required packages

.packages = c("here", "googlesheets4", "xlsx", "openxlsx", "readxl", "writexl", "tidyr", "plyr", "dplyr", "purrr", "ggplot2", "ggpubr", "ggpmisc", "ggrepel", "ggthemes", "ggpval", "ggforce", "cowplot", "lmerTest", "rstatix", "svglite","survminer", "survival", "RTCGA.clinical", "coxme", "gridExtra", "scales")

# install CRAN packages (if not already installed)

.inst <- .packages %in% installed.packages()

if(length(.packages[!.inst]) > 0) install.packages(.packages[!.inst])

# load packages into session

lapply(.packages, require, character.only=TRUE)

```

```{r, include=FALSE, warning=FALSE}

setwd(dirname(rstudioapi::getActiveDocumentContext()$path))

RawData <- read_excel("./RawData.xlsx", sheet= 1, skip=1, na = "")

RawData <- RawData %>% drop_na(Animal_ID)

SurvData <- RawData[,c("Animal_ID", "phenotype", "treatment", "Survivaltime_weeks", "death_category")]

SurvData[, c(1:3,5)] <- sapply(SurvData[, c(1:3,5)], as.factor)

SurvData[, 4] <- sapply(SurvData[,4], as.numeric)

colnames(SurvData)[which(names(SurvData) == "treatment")] <- "group"

SurvData$group <- factor(SurvData$group , levels=c("6-0 Silk", "7-0 Silk", "6-0 Prolene", "7-0 Prolene"))

colnames(SurvData)[which(names(SurvData) == "Survivaltime_weeks")] <- "survivaltime"

SurvData$vitalstatus <- NA

for(i in 1:nrow(SurvData)){

if(SurvData$death_category[i] == "died"){

SurvData$vitalstatus[i] <- 1

}else if(SurvData$death_category[i] == "sacrificed"){

SurvData$vitalstatus[i] <- 0

}else if(is.na(SurvData$death_category[i]) == TRUE){

SurvData$vitalstatus[i] <- NA

}else {}

}

grid.draw.ggsurvplot <- function(x){

survminer:::print.ggsurvplot(x, newpage = FALSE)}

################################################################################

filename <- tools::file_path_sans_ext(basename(rstudioapi::getActiveDocumentContext()$path))

Ranalysis_path <- paste0("./RESULTS.", filename)

if (!dir.exists(Ranalysis_path)) {dir.create(paste0("./RESULTS.", filename))}

outputDIR <- file.path(paste0("./RESULTS.", filename), as.character(Sys.Date()))

if (!dir.exists(outputDIR)) {dir.create(outputDIR)}

```

```{r}

SP <- list()

survival <- Surv(time = SurvData$survivaltime, event = SurvData$vitalstatus)

surv_fit <- survfit(survival ~ group, data = SurvData)

# Visualize with survminer

SP[[1]] <- ggsurvplot(

surv_fit, # survfit object with calculated statistics.

data = SurvData, # data used to fit survival curves.

risk.table = TRUE, # show risk table.

pval = TRUE, pval.size = 4, pval.coord = c(0.1, 0.1),

conf.int = FALSE, # show confidence intervals for point estimaes of survival curves.

xlim = c(0,12), # present narrower X axis, but not affect survival estimates.

break.time.by = 4, # break X axis in time intervals by 500.

ggtheme = theme_minimal(), # customize plot and risk table with a theme.

risk.table.y.text.col = T, # colour risk table text annotations.

risk.table.title = "",

#risk.table.y.text = FALSE, # show bars instead of names in text annotations in legend of risk table

tables.theme = theme_cleantable(), # clean theme for tables

palette = c("#66B2FF","#3333FF","#CC0066","#990000"), #"#66B2FF", "#66CC33", "#FF6600"

legend = "top",

legend.title = "",

legend.labs = c("6-0 Silk", "7-0 Silk", "6-0 Prolene", "7-0 Prolene"),

#title = "Kaplan-Meier Survival Analysis",

xlab = "survival time [weeks post TAC]",

ylab = "survival probability"

)

i=0.1

filename <- "SS3_Suture_Survival_all"

ggsave(filename =paste0(i, ".", filename, ".png"), path = outputDIR, device = "png",

SP[[1]], width = 10, height = 5, dpi = 300, units = "in")

print(SP[[1]])

```

```{r}

SurvData$group_material <- NA

for(i in 1:nrow(SurvData)){

if(SurvData$group[i] == "6-0 Prolene" | SurvData$group[i] == "7-0 Prolene"){

SurvData$group_material[i] <- "Prolene"

}else if(SurvData$group[i] == "6-0 Silk" | SurvData$group[i] == "7-0 Silk"){

SurvData$group_material[i] <- "Silk"

}else {}

}

SurvData$group_material <- factor(SurvData$group_material , levels=c("Silk", "Prolene"))

surv_fit2 <- survfit(survival ~ group_material, data = SurvData)

# Visualize with survminer

SP[[2]] <- ggsurvplot(

surv_fit2, # survfit object with calculated statistics.

data = SurvData, # data used to fit survival curves.

risk.table = TRUE, # show risk table.

pval = TRUE, pval.size = 4, pval.coord = c(0.1, 0.1),

conf.int = TRUE, # show confidence intervals for point estimaes of survival curves.

xlim = c(0,12), # present narrower X axis, but not affect survival estimates.

break.time.by = 4, # break X axis in time intervals by 500.

ggtheme = theme_minimal(), # customize plot and risk table with a theme.

risk.table.y.text.col = T, # colour risk table text annotations.

risk.table.title = "",

#risk.table.y.text = FALSE, # show bars instead of names in text annotations in legend of risk table

tables.theme = theme_cleantable(), # clean theme for tables

palette = c("#3333FF","#990000"), #"#66B2FF", "#66CC33", "#FF6600"

legend = "top",

legend.title = "",

legend.labs = c("Silk","Prolene"),

xlab = "survival time [weeks post TAC]",

ylab = "survival probability"

)

i=0.2

filename <- "SS3_Suture_Survival_material"

ggsave(filename =paste0(i, ".", filename, ".png"), path = outputDIR, device = "png",

SP[[2]], width = 10, height = 5, dpi = 300, units = "in")

print(SP[[2]])

```

```{r}

SurvData$group_size <- NA

for(i in 1:nrow(SurvData)){

if(SurvData$group[i] == "7-0 Silk" | SurvData$group[i] == "7-0 Prolene"){

SurvData$group_size[i] <- "7-0"

}else if(SurvData$group[i] == "6-0 Silk" | SurvData$group[i] == "6-0 Prolene"){

SurvData$group_size[i] <- "6-0"

}else {}

}

SurvData$group_size <- factor(SurvData$group_size , levels=c("6-0", "7-0"))

surv_fit3 <- survfit(survival ~ group_size, data = SurvData)

# Visualize with survminer

SP[[3]] <- ggsurvplot(

surv_fit3, # survfit object with calculated statistics.

data = SurvData, # data used to fit survival curves.

risk.table = TRUE, # show risk table.

pval = TRUE, pval.size = 4, pval.coord = c(0.1, 0.1),

conf.int = TRUE, # show confidence intervals for point estimaes of survival curves.

xlim = c(0,12), # present narrower X axis, but not affect survival estimates.

break.time.by = 4, # break X axis in time intervals by 500.

ggtheme = theme_minimal(), # customize plot and risk table with a theme.

risk.table.title = "",

risk.table.y.text.col = T, # colour risk table text annotations.

#risk.table.y.text = FALSE, # show bars instead of names in text annotations in legend of risk table

tables.theme = theme_cleantable(), # clean theme for tables

palette = c("grey","black"), #"#66B2FF", "#66CC33", "#FF6600"

legend = "top",

legend.title = "",

legend.labs = c("6-0", "7-0"),

#title = "Kaplan-Meier Survival Analysis",

xlab = "survival time [weeks post TAC]",

ylab = "survival probability"

)

i=0.3

filename <- "SS3_Suture_Survival_size"

ggsave(filename =paste0(i, ".", filename, ".png"), path = outputDIR, device = "png",

SP[[3]], width = 10, height = 5, dpi = 300, units = "in")

write_xlsx(SurvData, paste0(outputDIR, "/", i, ".",filename, ".xlsx"))

print(SP[[3]])

```

```{r}

SP[[1]]$plot <- SP[[1]]$plot + labs(tag="A")

SP[[2]]$plot <- SP[[2]]$plot + labs(tag="B")

SP[[3]]$plot <- SP[[3]]$plot + labs(tag="C")

figure <- arrange_ggsurvplots(SP,

labels = c("A", "B", "C"),

ncol = 3, nrow = 1)

i=0.0

filename <- "SS3_Suture_Survival_summary"

ggsave(filename =paste0(i, ".", filename, ".png"), path = outputDIR, device = "png",

figure, width = 15, height = 5, dpi = 300, units = "in")

```
