# Supplementary figures and images for: The type of suture material affects transverse aortic constriction-induced heart failure development in mice: a repeated measures correlation analysis
Source: Front Cardiovasc Med. 2023 Sep 19;10:1242763. doi: 10.3389/fcvm.2023.1242763 (PMC10546326; doi:10.3389/fcvm.2023.1242763)

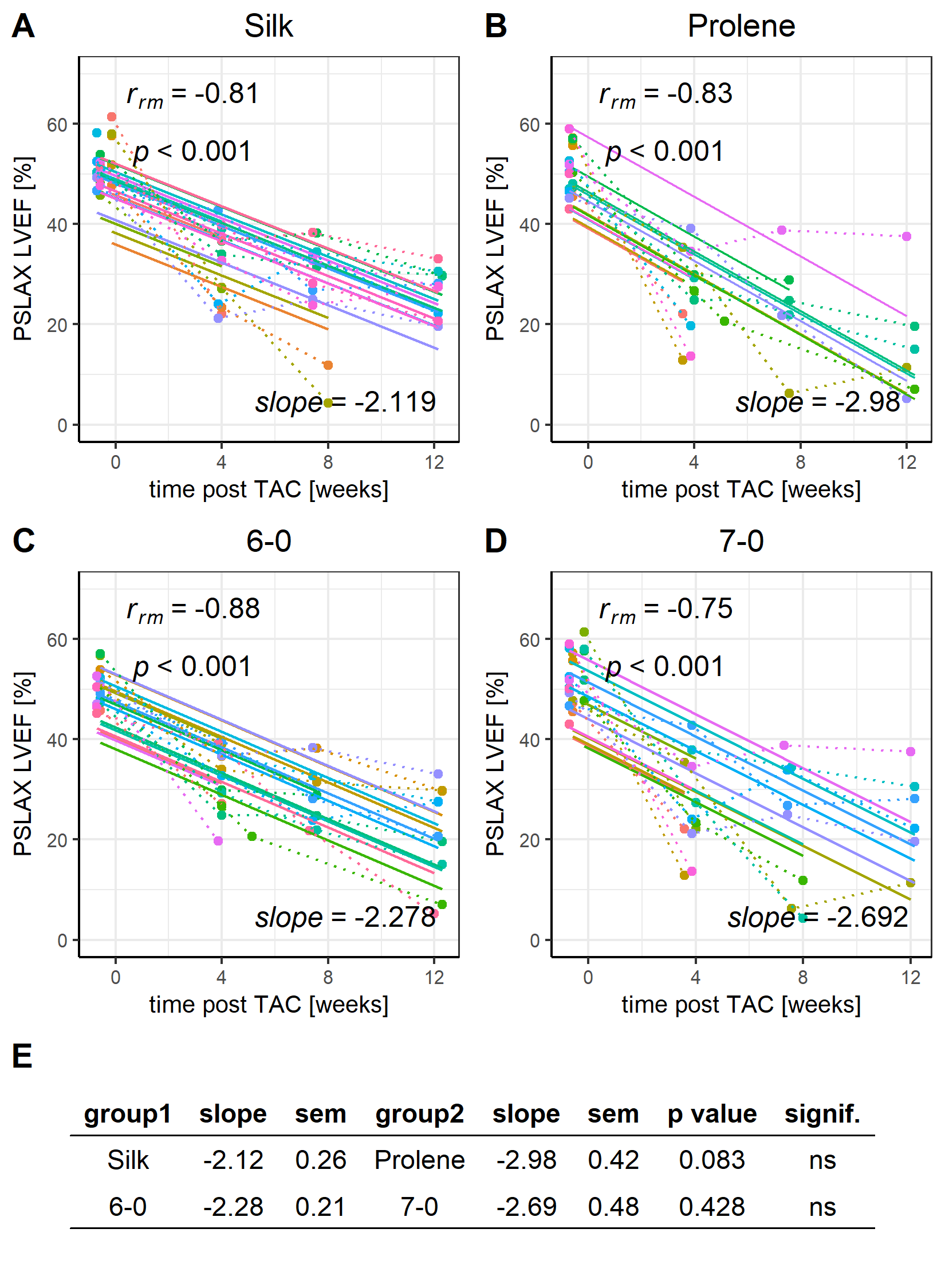

Supplement: Supplementary file 2 [file Datasheet2.zip › FigS10_RmCorr_LVEF_longAxis.tif]

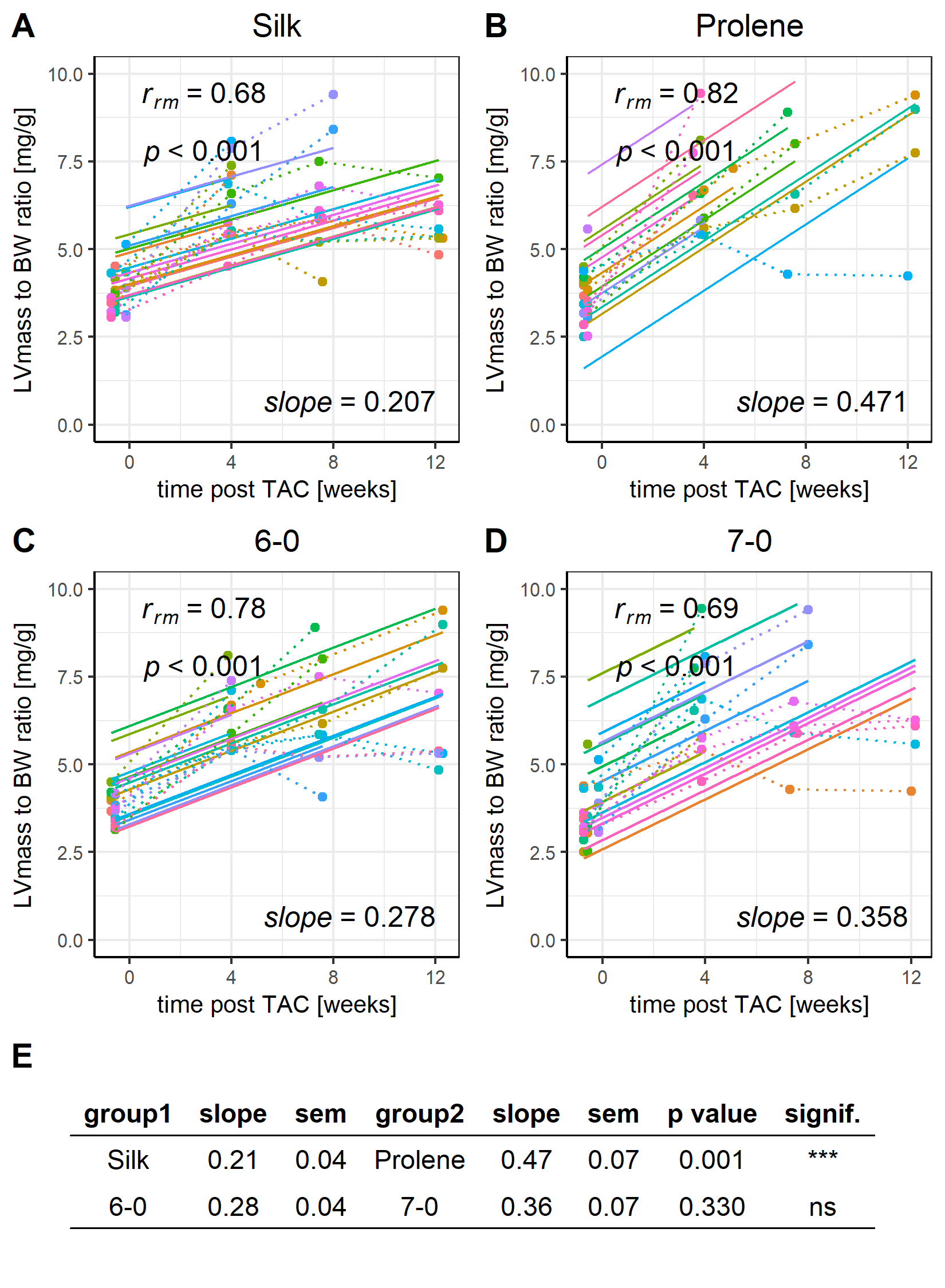

Supplement: Supplementary file 2 [file Datasheet2.zip › FigS11_RmCorr_HW_BW.tif]

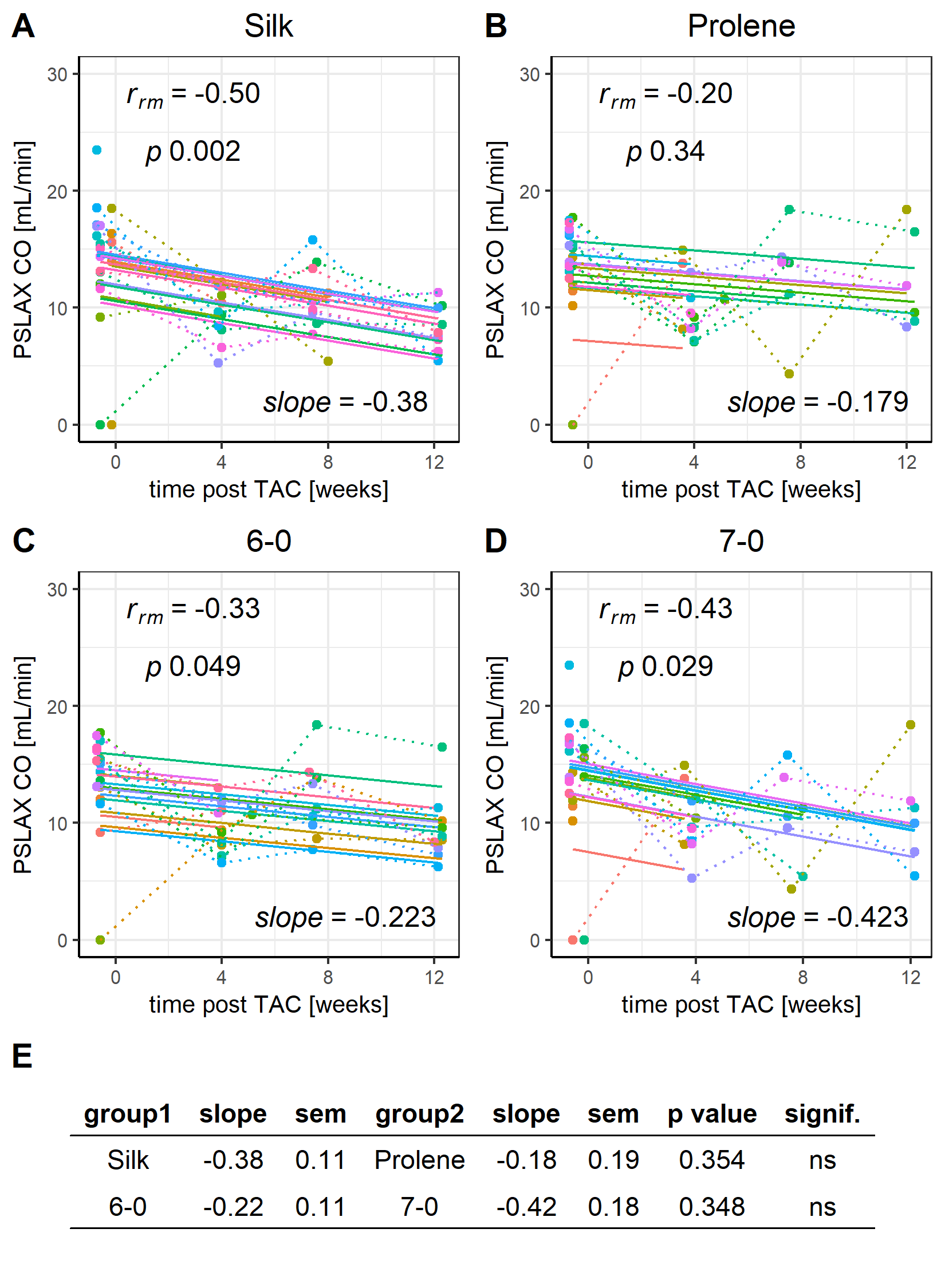

Supplement: Supplementary file 2 [file Datasheet2.zip › FigS12_RmCorr_CO_mLpmin.tif]

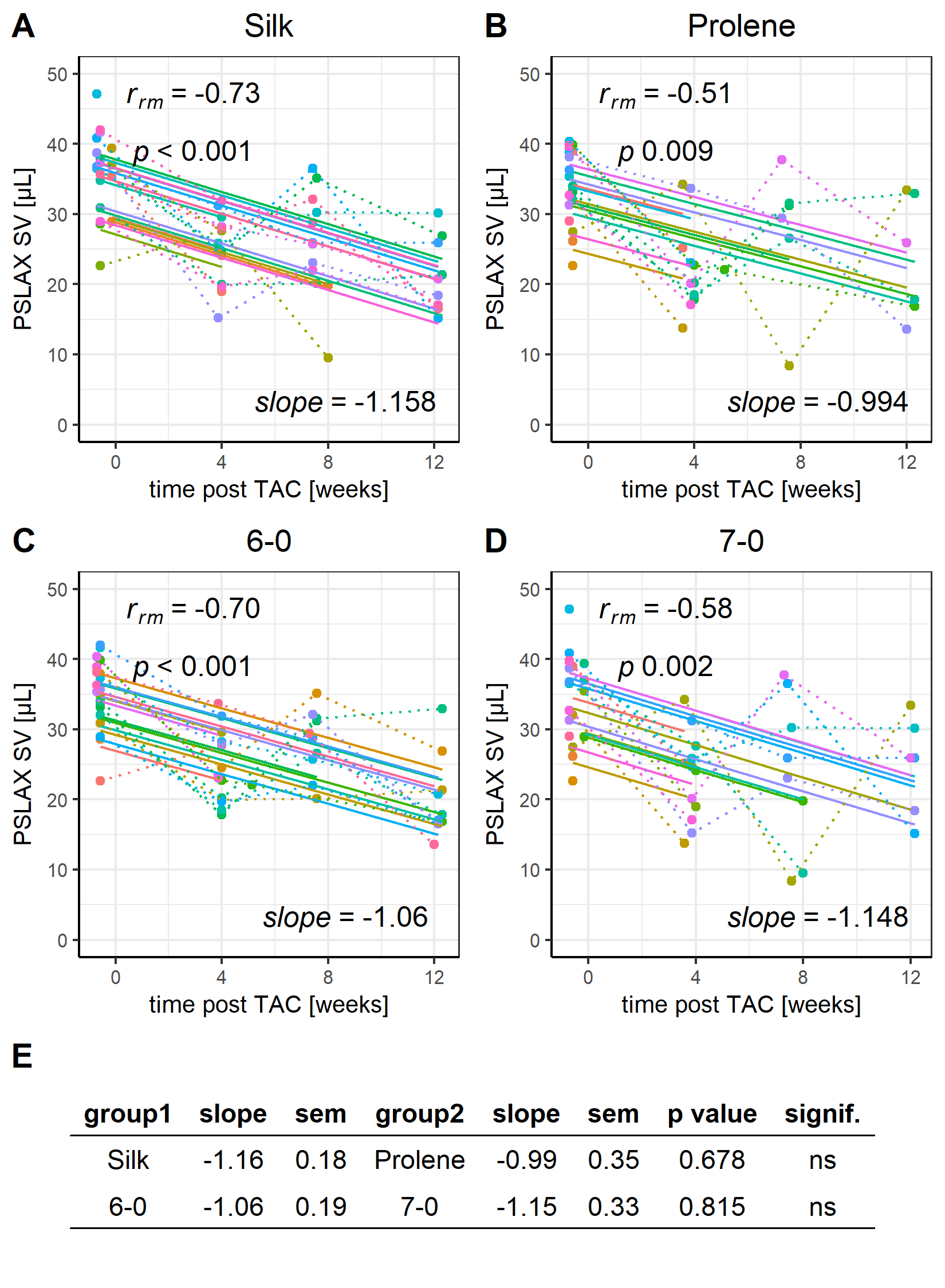

Supplement: Supplementary file 2 [file Datasheet2.zip › FigS13_RmCorr_SV_uL.tif]

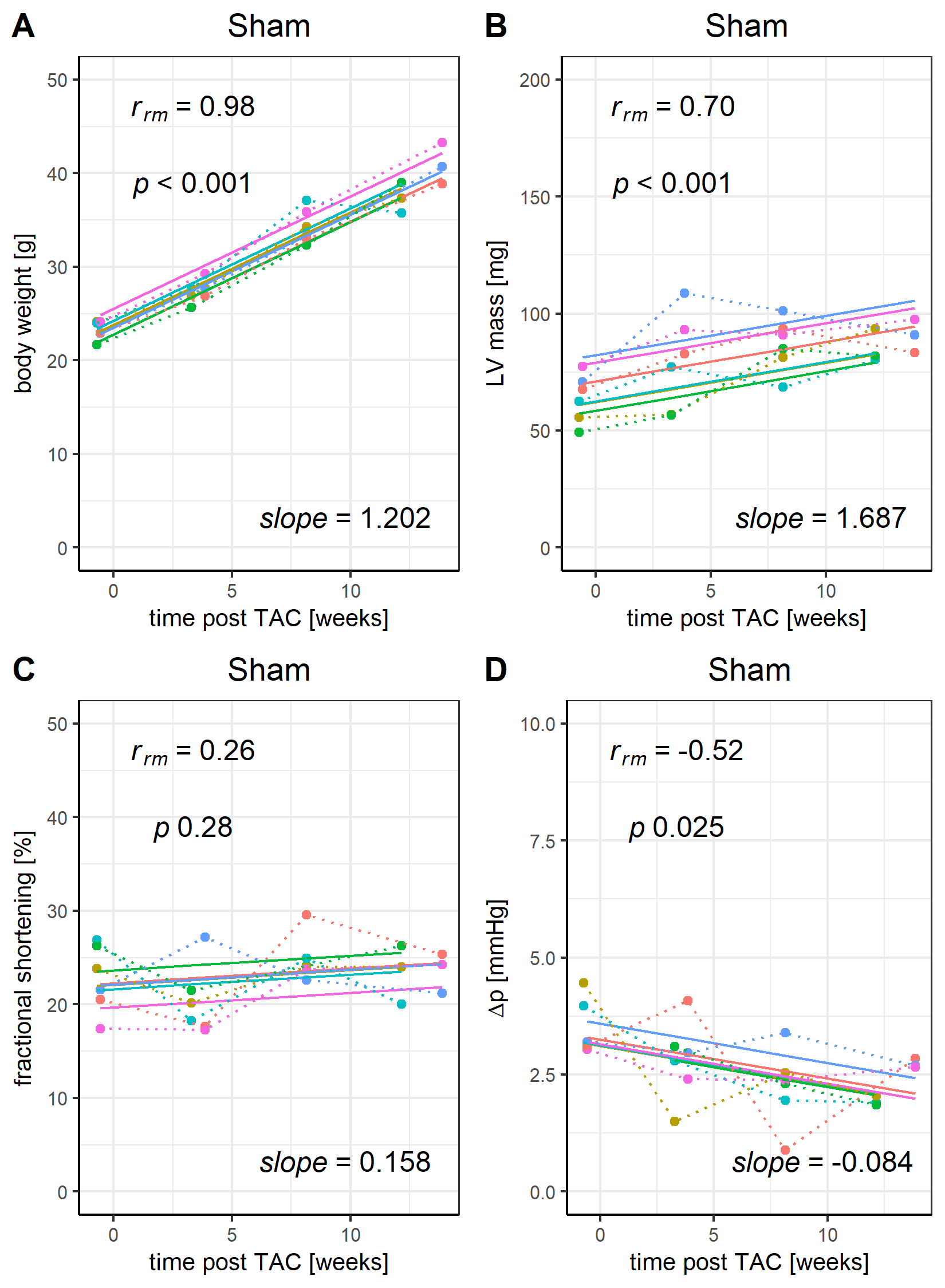

Supplement: Supplementary file 2 [file Datasheet2.zip › FigS14_RmCorr_sham.tif]

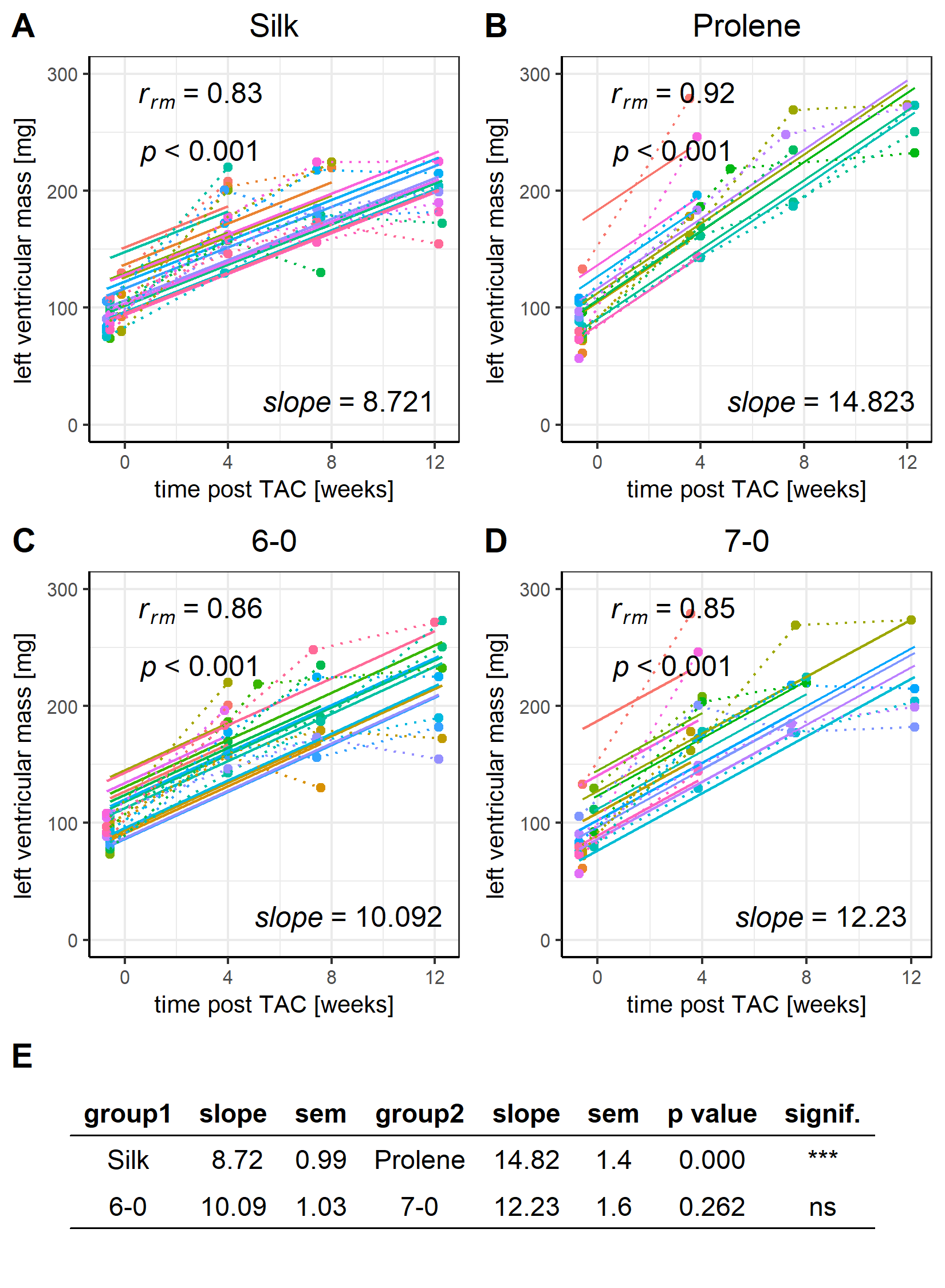

Supplement: Supplementary file 2 [file Datasheet2.zip › FigS15_RmCorr_LVmass_corr_no385.tif]

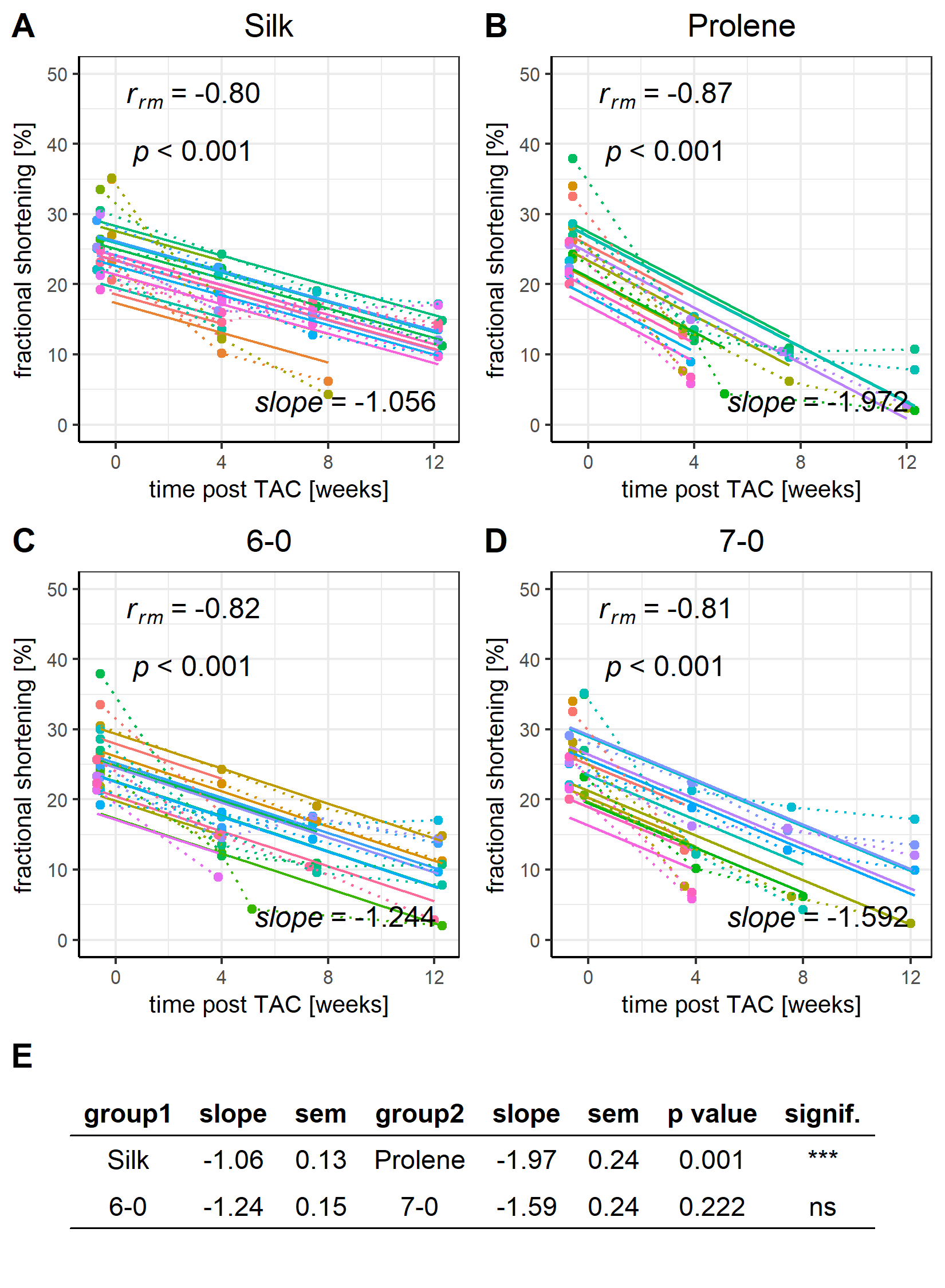

Supplement: Supplementary file 2 [file Datasheet2.zip › FigS16_RmCorr_FS_no385.tif]

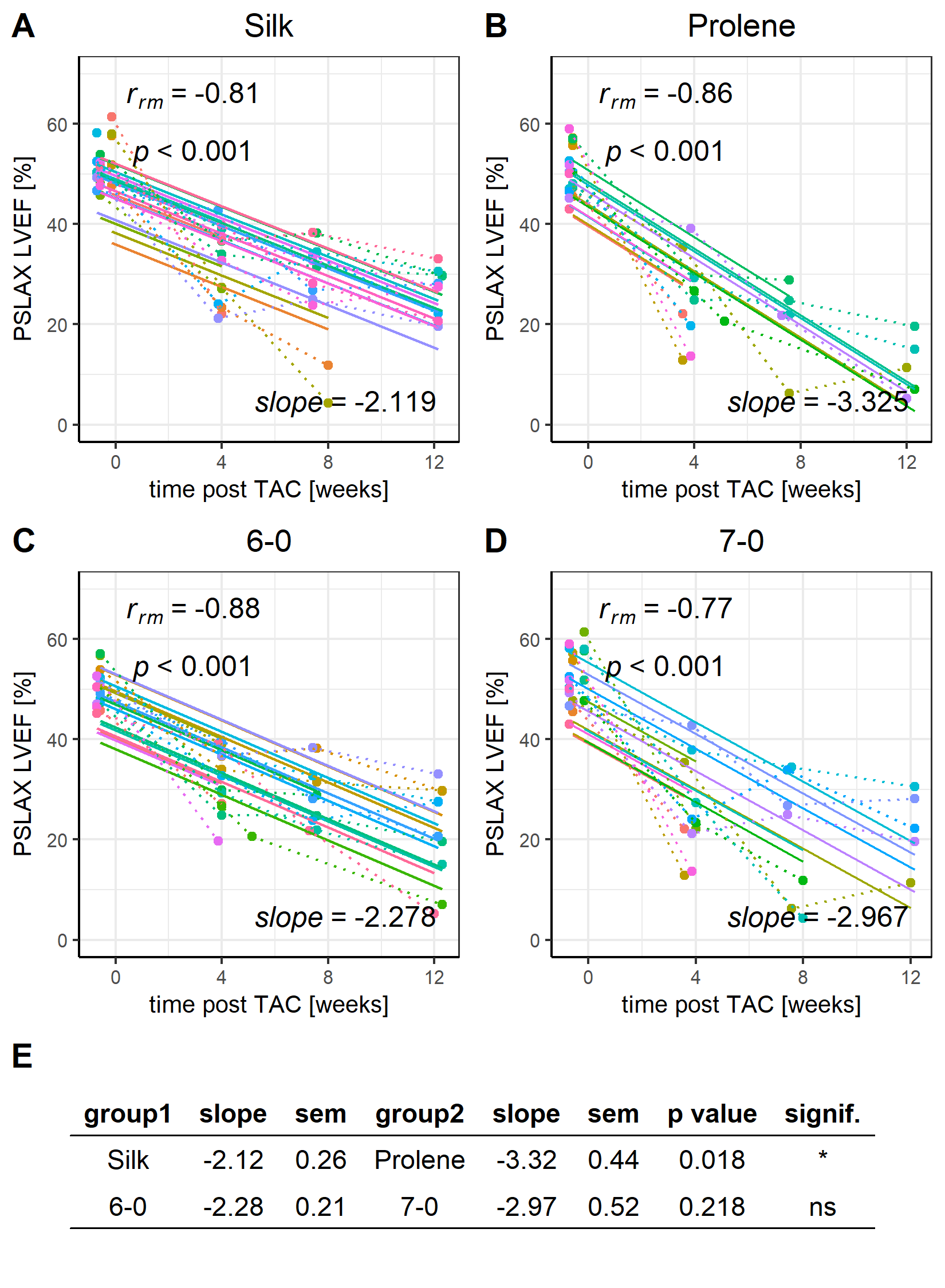

Supplement: Supplementary file 2 [file Datasheet2.zip › FigS17_RmCorr_LVEF_longAxis_no385.tif]

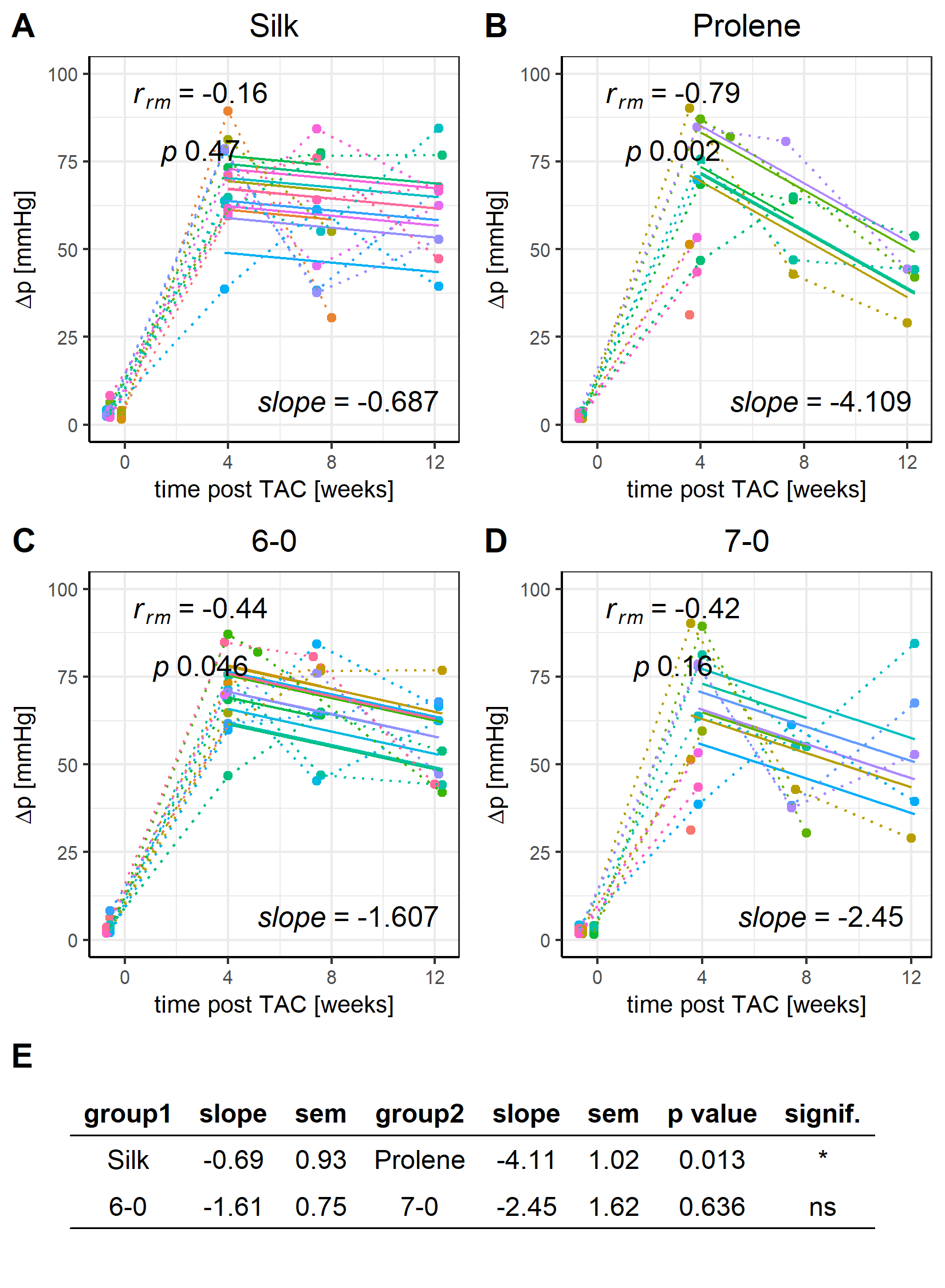

Supplement: Supplementary file 2 [file Datasheet2.zip › FigS18_RmCorr_pgrad_no385.tif]

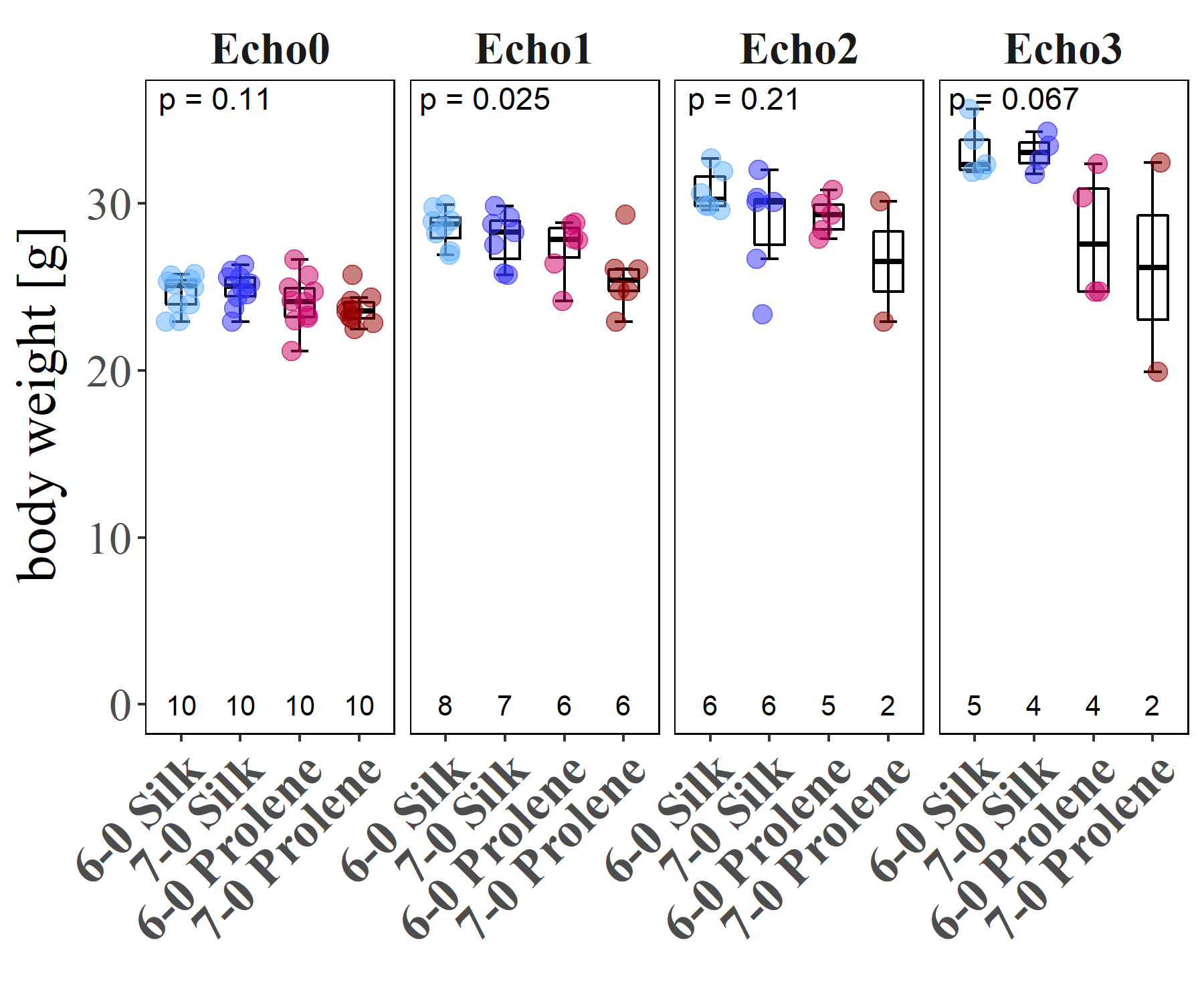

Supplement: Supplementary file 2 [file Datasheet2.zip › FigS1_Anova_all_BW.tif]

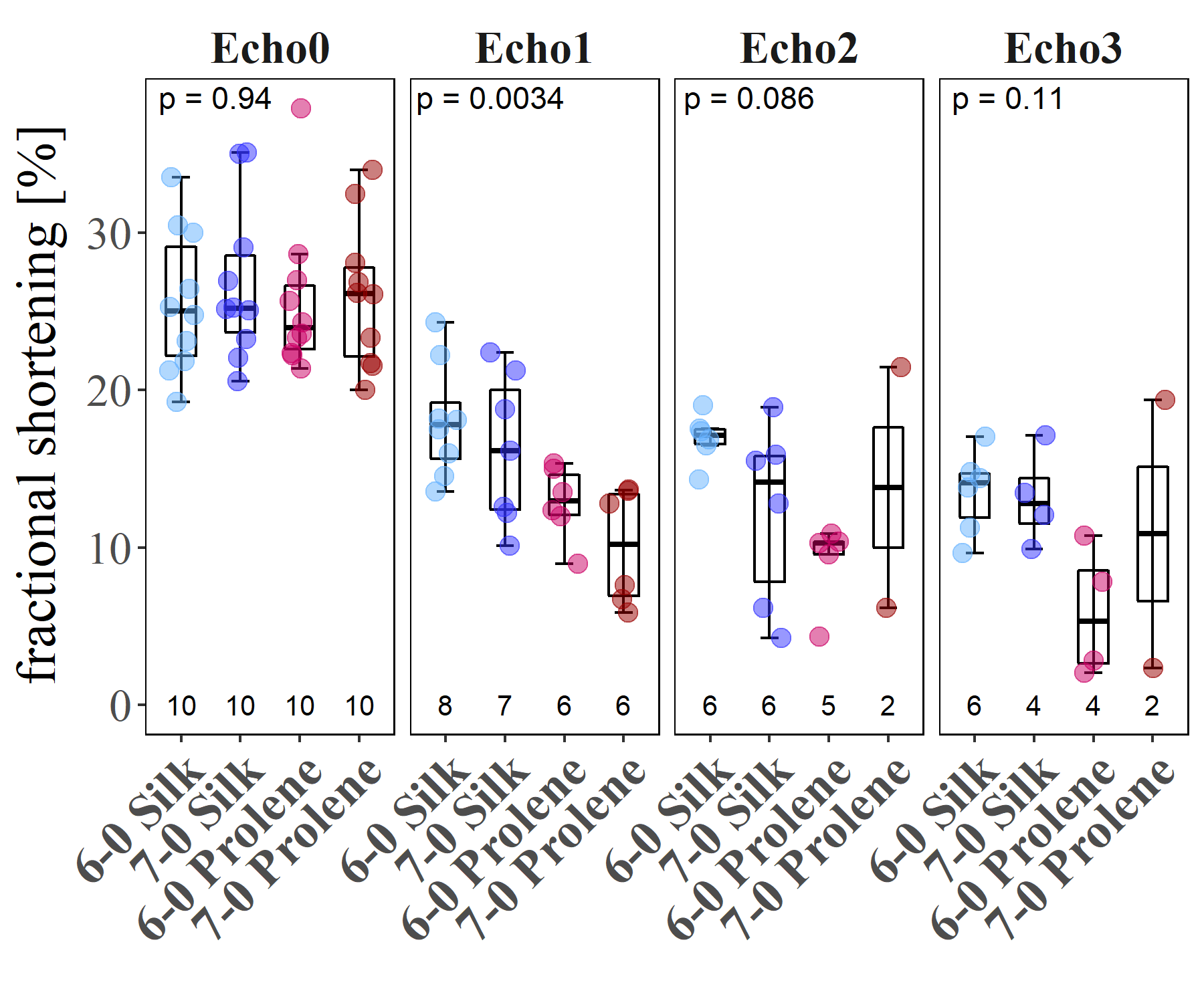

Supplement: Supplementary file 2 [file Datasheet2.zip › FigS2_Anova_all_FS.tif]

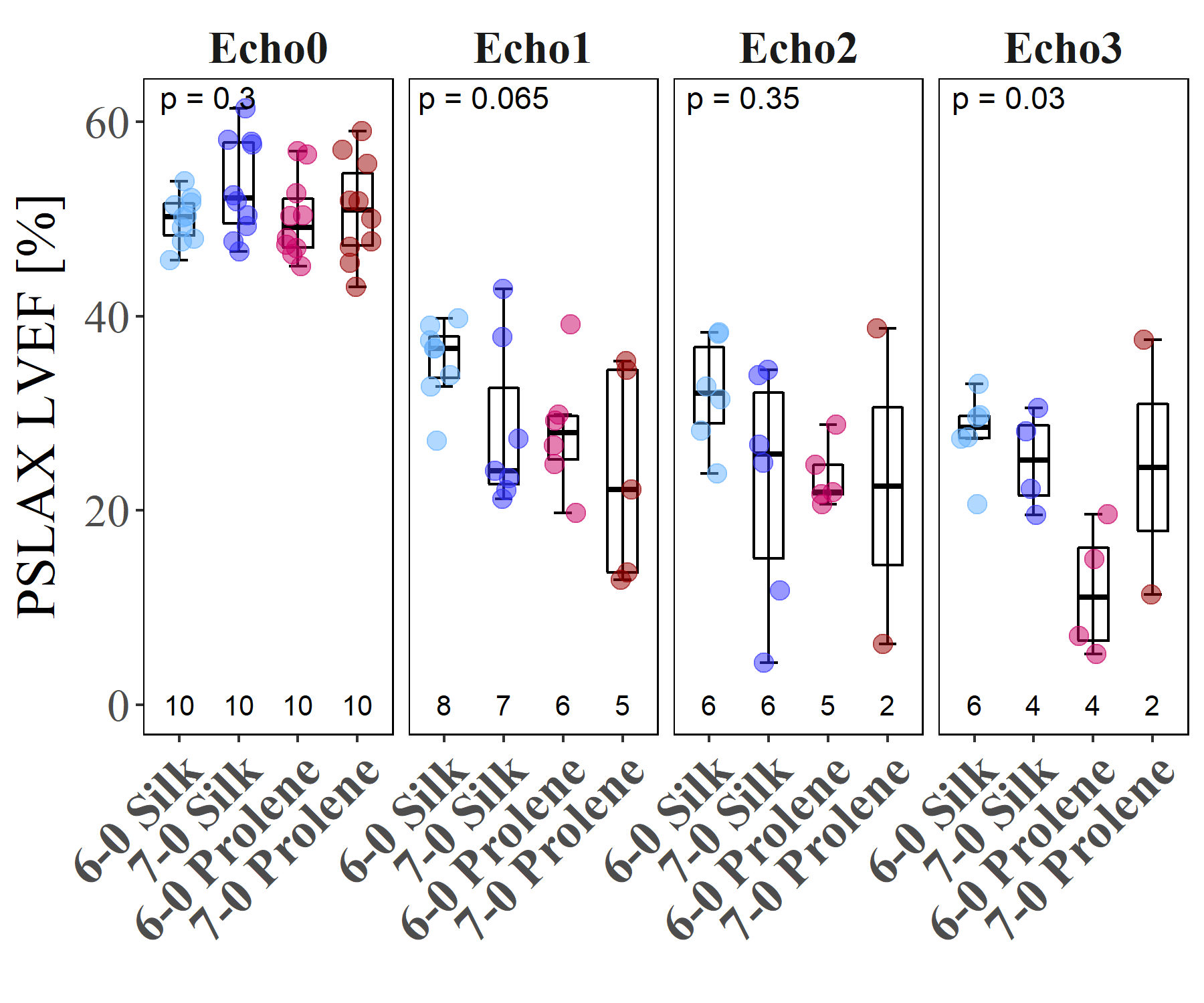

Supplement: Supplementary file 2 [file Datasheet2.zip › FigS3_Anova_all_LVEF_longAxis.tif]

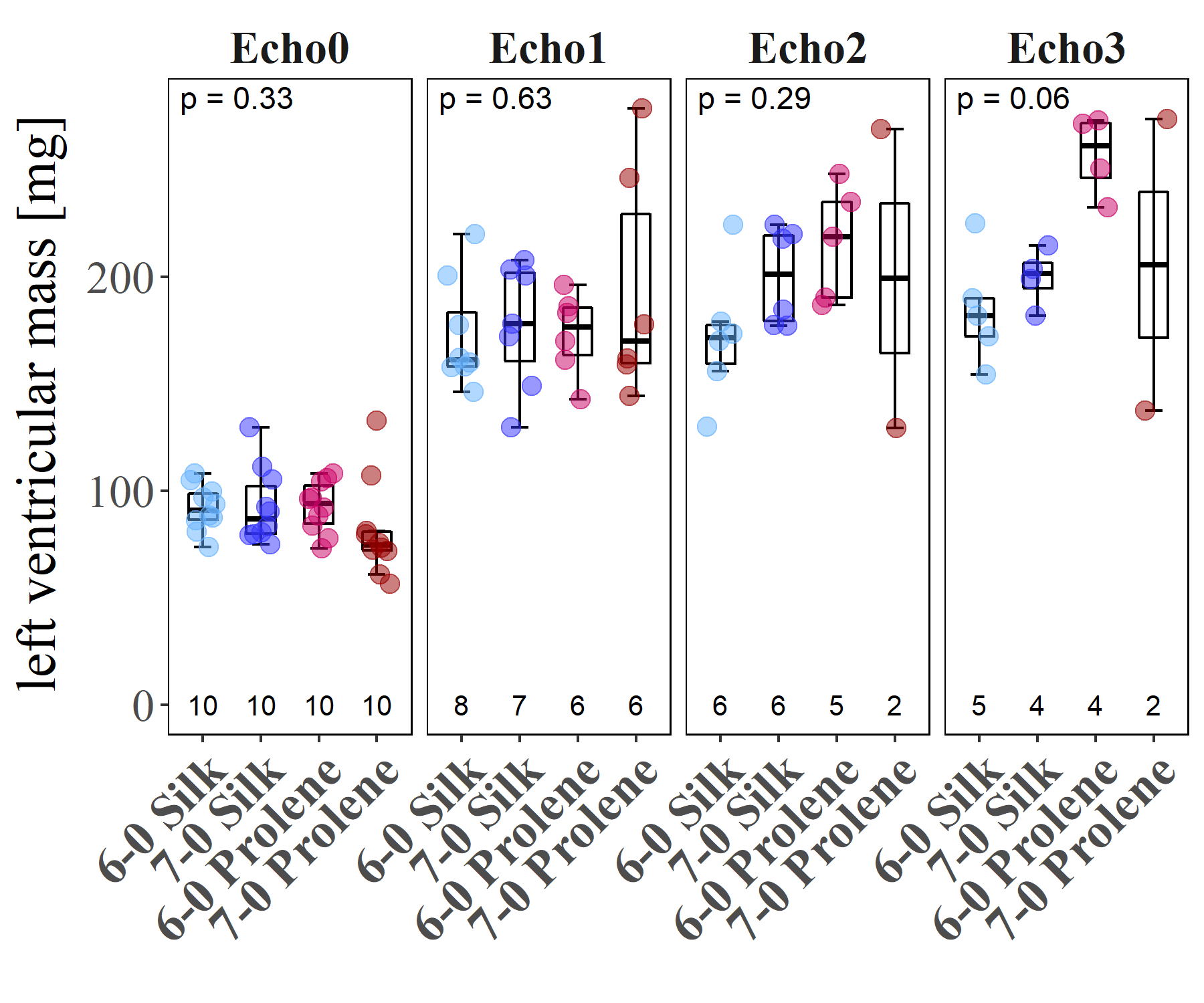

Supplement: Supplementary file 2 [file Datasheet2.zip › FigS4_Anova_all_LVmass_corr.tif]

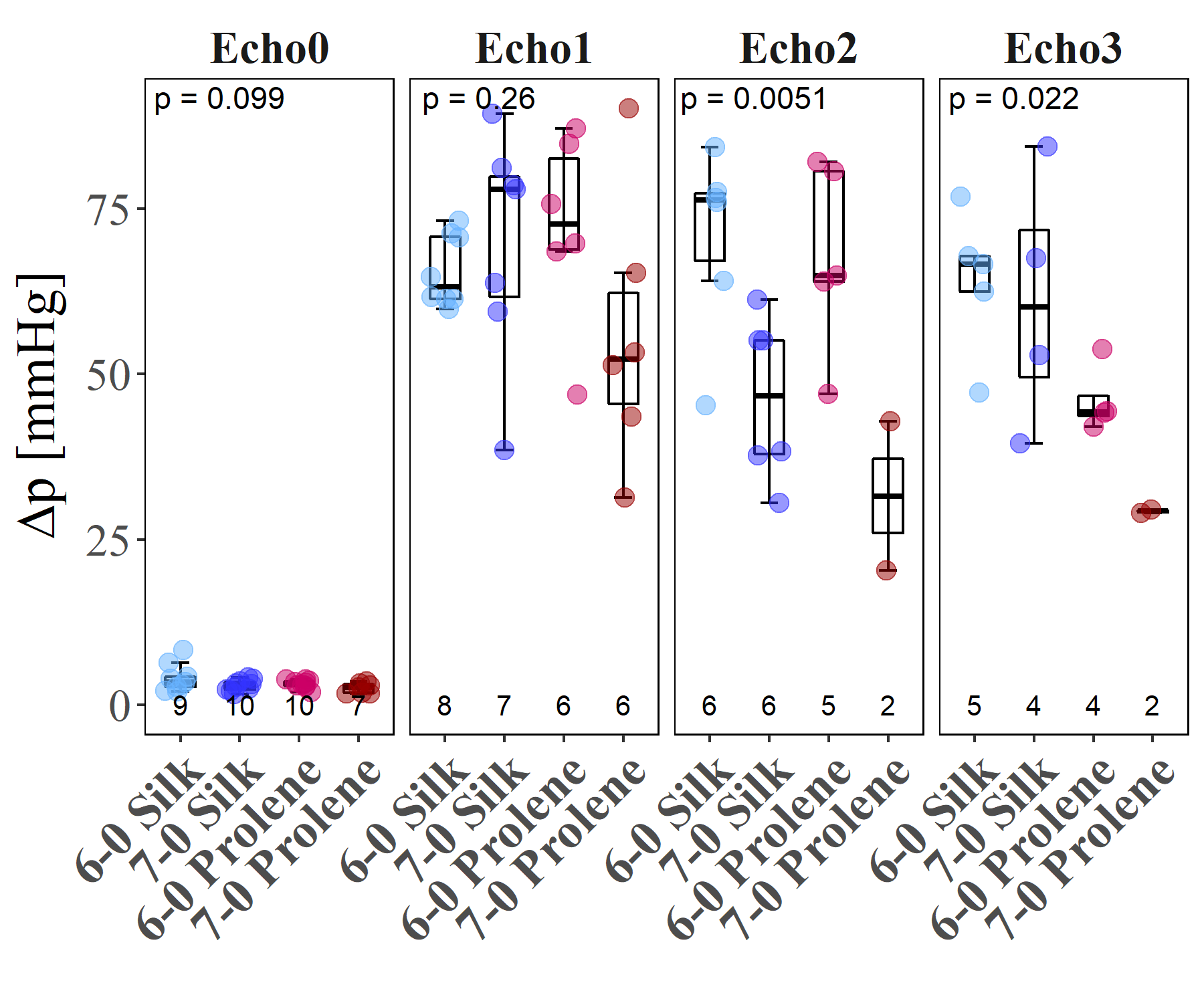

Supplement: Supplementary file 2 [file Datasheet2.zip › FigS5_Anova_all_pgrad.tif]

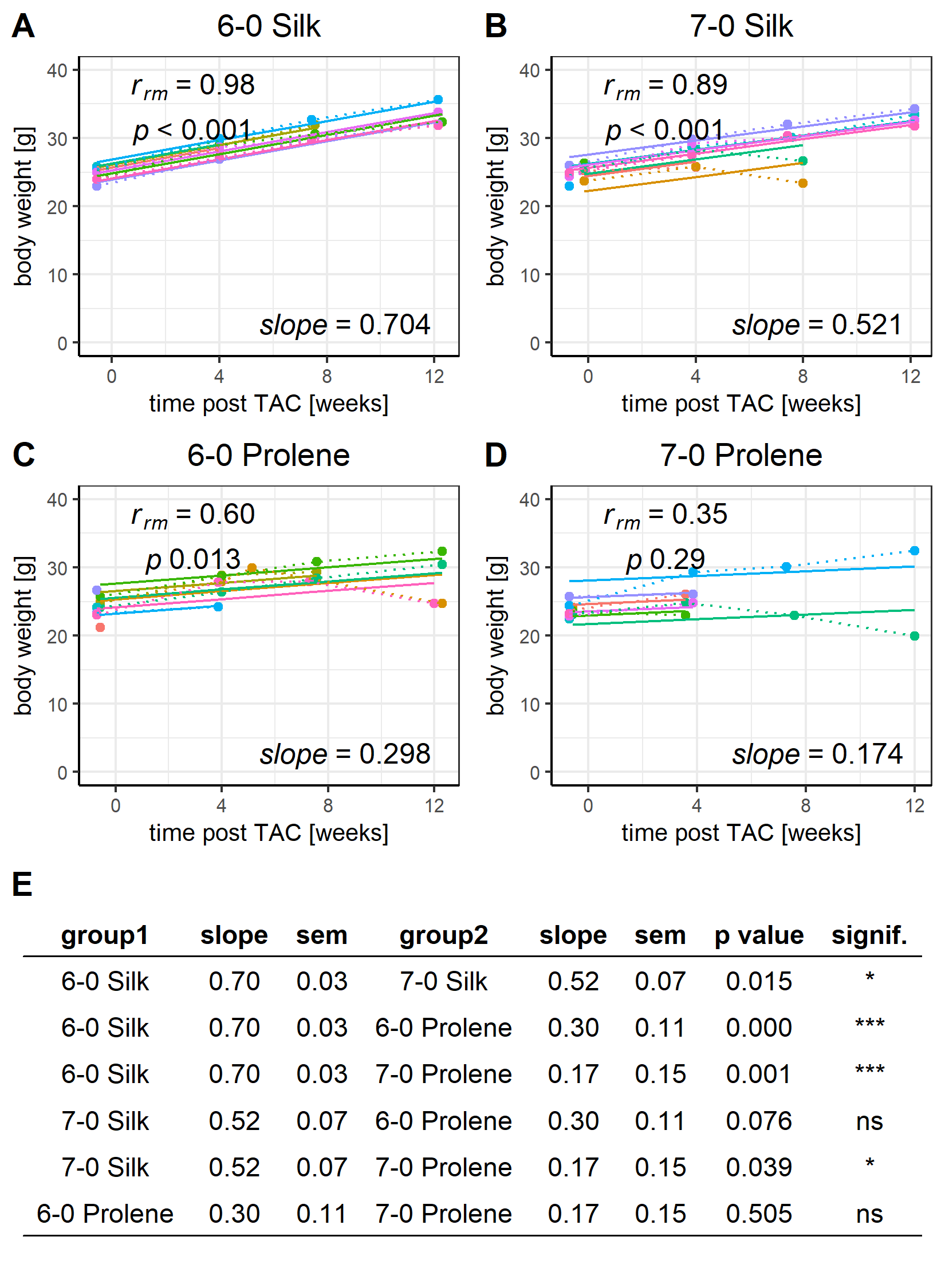

Supplement: Supplementary file 2 [file Datasheet2.zip › FigS6_RmCorr_all_BW.tif]

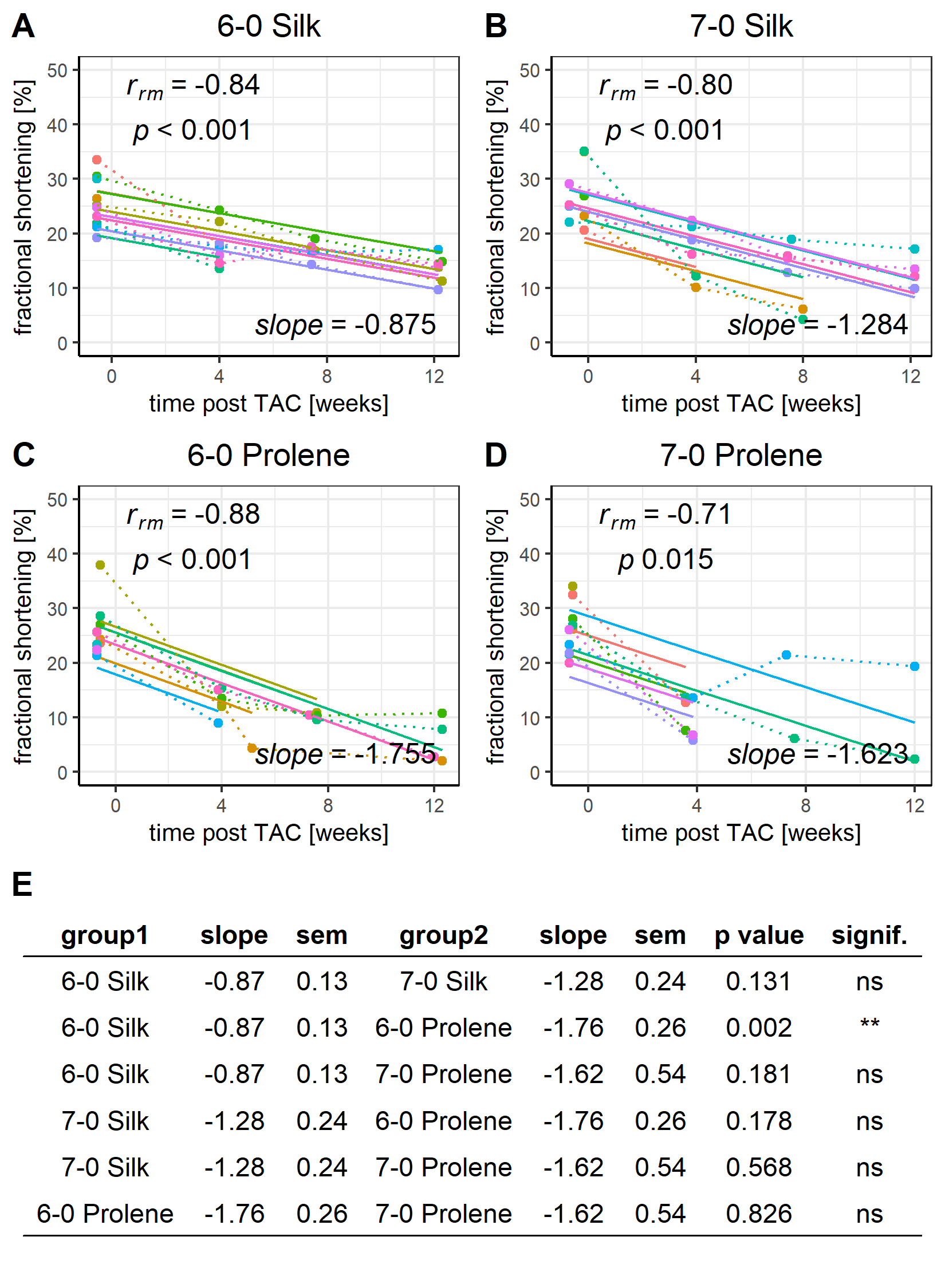

Supplement: Supplementary file 2 [file Datasheet2.zip › FigS7_RmCorr_all_FS.tif]

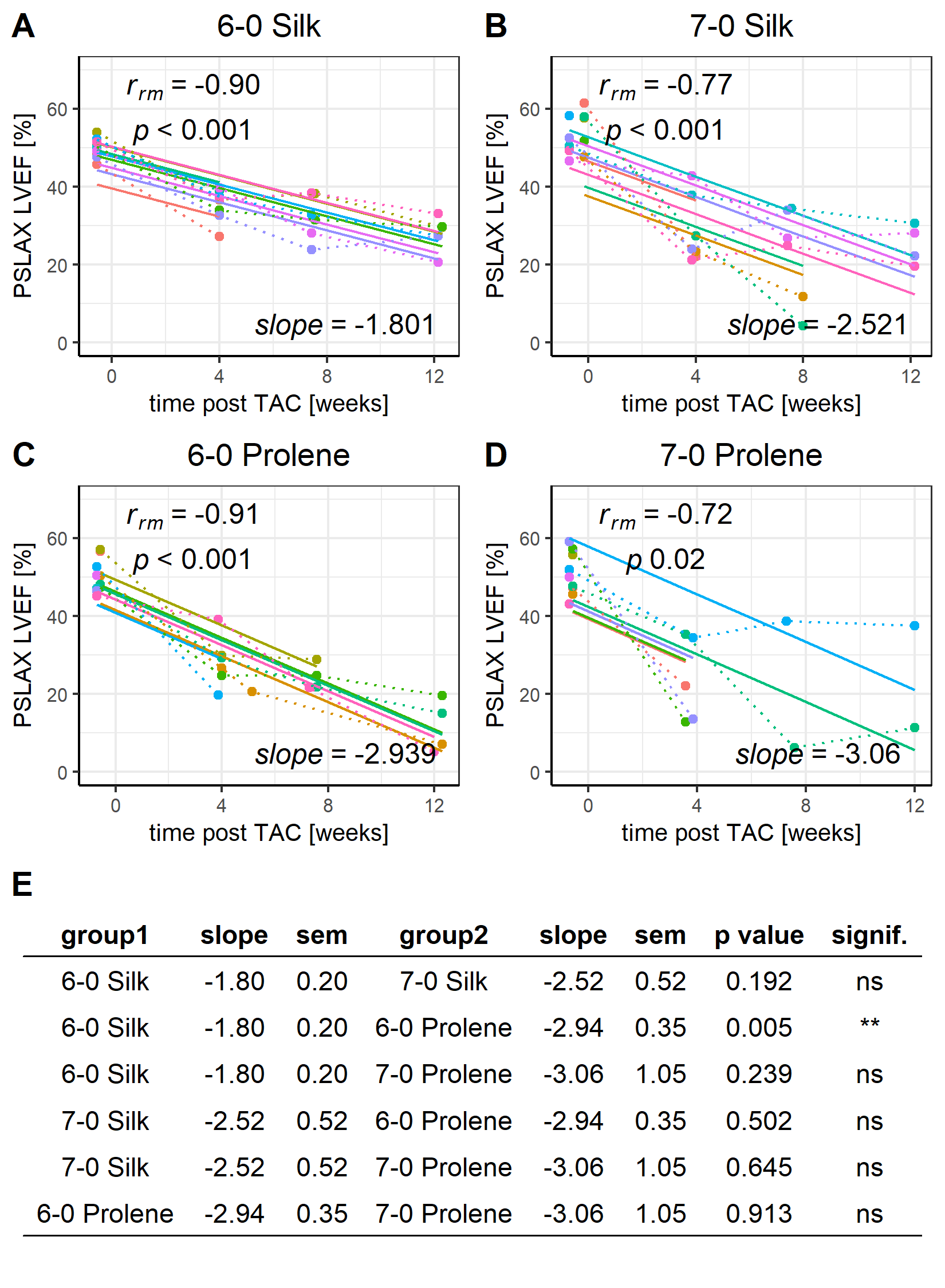

Supplement: Supplementary file 2 [file Datasheet2.zip › FigS8_RmCorr_all_LVEF_longAxis.tif]

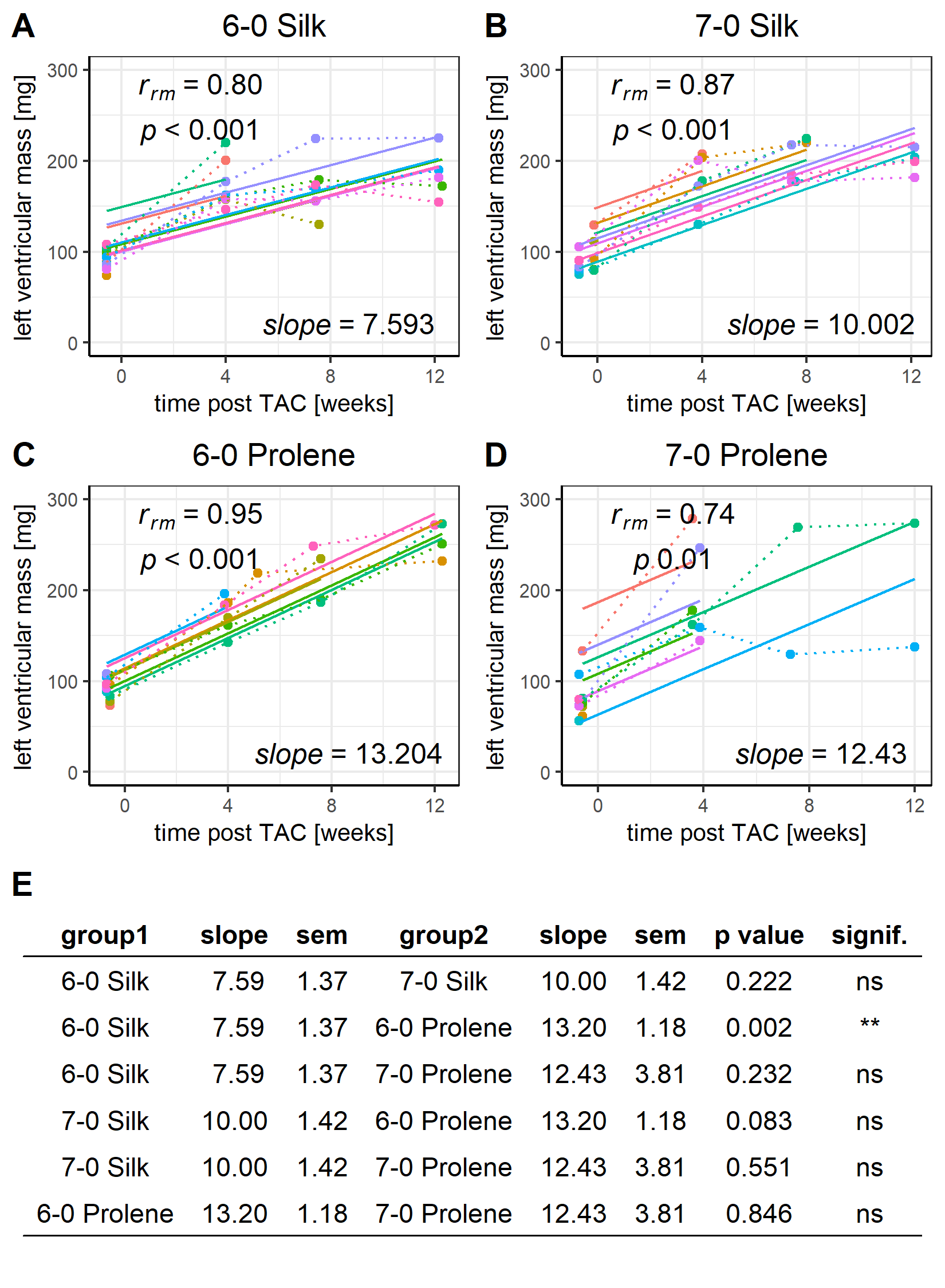

Supplement: Supplementary file 2 [file Datasheet2.zip › FigS9_RmCorr_all_LVmass_corr.tif]
